# Corrigendum: Analysis of tick-borne encephalitis virus-induced host responses in human cells of neuronal origin and interferon-mediated protection

**DOI:** 10.1099/jgv.0.001109

**Published:** 2018-08-01

**Authors:** Martin Selinger, Gavin S. Wilkie, Lily Tong, Quan Gu, Esther Schnettler, Libor Grubhoffer, Alain Kohl

**Affiliations:** ^1^​ Institute of Parasitology, Biology Centre of the Academy of Sciences of the Czech Republic, Branišovská 31, 370 05 České Budějovice, Czech Republic; ^2^​ Faculty of Science, University of South Bohemia in České Budějovice, Branišovská 31, 370 05 České Budějovice, Czech Republic; ^3^​ MRC-University of Glasgow Centre for Virus Research, Glasgow G61 1QH, Scotland, UK; ^†^​ Present address: Bernhard Nocht Institute for Tropical Medicine, Bernhard-Nocht-Str. 74, 20359 Hamburg, Germany.

**Keywords:** host response, neuronal cells, transcriptome analysis, interferon, tick-borne encephalitis virus

Due to a mistake in data set export, an error was introduced by which CTD-2328D6.1 was mistakenly switched to RNA28S5 in Table S5, leading to this gene wrongly being mentioned in the text on several occasions, and errors in Fig. 4.

The corrected Table S5 is shown in the supplementary material.

On page 2046, section‚ ‘Host response-associated genes, including type III IFNs, are activated upon TBEV infection of DAOY cells’, the first appearance of ‘RNA28S5’ should be changed to ‘CTD-2328D6.1’ and the final appearance of ‘RNA28S5’ should be removed from the following section of text.

‘RNA28S5, RN7SL2, NOTCH3, COL1A1, BCL9L, BCORL1, POLR2A, FAM71D, IGF2, RN7SL3 and HSPG2 were found to be the most strongly down-regulated genes (fold-change >2.5; Fig. 4 and Table S5). Other than protein-coding genes, a number of non-coding RNAs were also identified as being differentially expressed upon TBEV infection, as shown in Fig. 4(b). However, of these, RN7SL2, RN7SL3 and RNA28S5 are the only RNA genes with known functions.’

This section should read as follows:

‘CTD-2328D6.1, RN7SL2, NOTCH3, COL1A1, BCL9L, BCORL1, POLR2A, FAM71D, IGF2, RN7SL3 and HSPG2 were found to be the most strongly down-regulated genes (fold-change >2.5; Fig. 4 and Table S5). Other than protein-coding genes, a number of non-coding RNAs were also identified as being differentially expressed upon TBEV infection, as shown in Fig. 4(b). However, of these, RN7SL2 and RN7SL3 are the only RNA genes with known functions.’

On page 2052, right-hand column, paragraph 2, the first occurrence of ‘RNA28S5’ should be removed and the final appearance of ‘RNA28S5’ should be changed to ‘CTD-2328D6.1’ in the following section of text.

‘Downregulation of effectors involved in either transcription (POLR2A) or translation (RNA28S5, RN7SL2, RN7SL3) suggests a possible TBEV-driven transcriptional or translational shut off in host cells. Both transcriptional and translational shut off are well-documented phenomena [63, 64]. Similar rates of RNA28S5, NOTCH3, COL1A1, BCL9L, BCOR1, POLR2A, FAM71D, IGF2 and HSPG2 down-regulation were also evident in IFN-β-pre-treated cells infected with TBEV, where significantly lower viral titres were determined.’

This section should read as follows:

‘Downregulation of effectors involved in either transcription (POLR2A) or translation (RN7SL2, RN7SL3) suggests a possible TBEV-driven transcriptional or translational shut off in host cells. Both transcriptional and translational shut off are well-documented phenomena [63, 64]. Similar rates of CTD-2328D6.1, NOTCH3, COL1A1, BCL9L, BCOR1, POLR2A, FAM71D, IGF2 and HSPG2 down-regulation were also evident in IFN-β-pre-treated cells infected with TBEV, where significantly lower viral titres were determined.’

An error also occured in Table S3 due to a formatting mistake. The following changes are shown in the updated supplementary material:

‘3.01’ on page 1 in column ‘IFN-β’ was corrected to ‘MARC1’.

‘9.09’ on page 10 in column ‘TBEV’ was corrected to ‘SEPT9’.

‘3.04’ on page 12 in column ‘TBEV’ was corrected to ‘MARCH4’.

‘9.09’ on page 14 in column ‘IFN-β + TBEV’ was corrected to ‘SEPT9’.

‘3.03’ on page 20 in column ‘IFN-β + TBEV’ was corrected to ‘MARCH3’.

Fig. 4 also required a correction in the heatmap and four genes that were in panel (a) should only be present in panel (b), with RNA28S5 deleted from the figure. The corrected Fig. 4 is shown above.

**Fig. 4.**
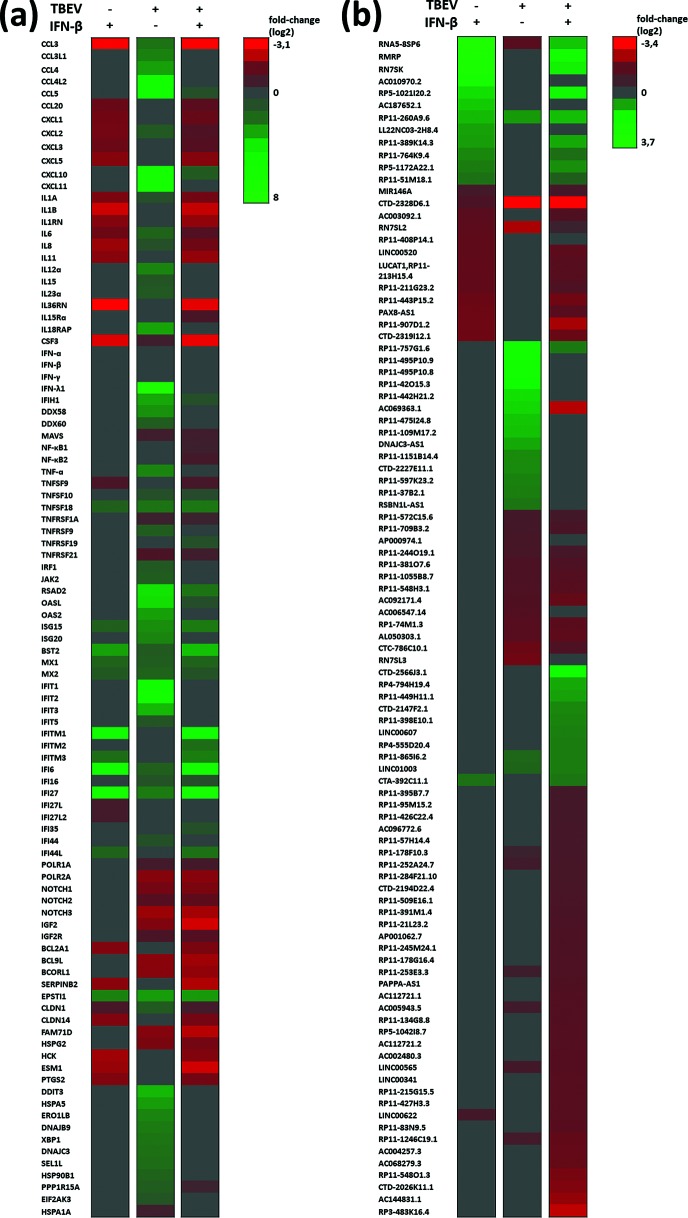
Overview of selected differentially expressed genes. DAOY cells were pre-treated with IFN-β (10 ng ml^−1^) and/or infected with TBEV (m.o.i. 5) after 12 h. Three independent biological replicates were included for each of the combinations [untreated mock cells (control); IFN-β-treated mock cells; untreated cells infected with TBEV; IFN-β-pre-treated cells infected with TBEV]. Total cellular RNA was isolated at 24 h p.i. and used for transcriptome analysis. (a) List of selected protein-coding genes identified to be differentially expressed in at least one of the combinations over control (Benjamini Hochberg *P*-value ≤0.05 and fold change >1.5 or <−1.5; down-regulated in red and up-regulated in green). To emphasize the up-regulation of IFN-λ1, information for transcripts of IFN-ɑ, IFN-β and IFN-γ was also included. (b) List of selected non-coding genes identified to be differentially expressed in at least one of the combinations over control (Benjamini Hochberg *P*-value ≤0.05 and fold change >1.5 or <−1.5; down-regulated in red and up-regulated in green).

The authors apologise for any inconvenience caused.

## Supplementary Data

Supplementary File 1

