# Supplementary material for: Corrigendum: Analysis of tick-borne encephalitis virus-induced host responses in human cells of neuronal origin and interferon-mediated protection
Source: J Gen Virol. 2018 Aug 1;99(8):1147–9. doi: 10.1099/jgv.0.001109 (PMC6171712; doi:10.1099/jgv.0.001109)

<u>Supplementary material - Tables S1-S8, Fig. S1, Fig. S2</u>

Table S1: List of selected glial cell markers and number of mapped reads in DAOY cells. Each value represents an average number of reads from three independent biological replicates used for transcriptome analysis.

accession number	sample ID	TBEV	+	+	-	-
		IFN-β	+	-	+	-
NM_003380.3	vimentin (VIM)		154398	144583	227636	142538
NM_000610.3	CD44 molecule (Indian blood group) (CD44), transcript variant 1		29096	29513	44064	33944
NM_001008228.2	myelin oligodendrocyte glycoprotein (MOG), transcript variant alpha3		22706	24121	30079	23299
NM_206809.3	myelin oligodendrocyte glycoprotein (MOG), transcript variant alpha1		22706	24121	30079	23299
NM_004171.3	Solute carrier family 1 member 2 (SLC1A2), transcript variant 1		9588	10268	12938	9645
NM_004124.2	glia maturation factor beta (GMFB)		6875	6364	8076	6476
NM_023110.2	fibroblast growth factor receptor 1 (FGFR1), transcript variant 1		6823	7057	10580	7450
NM_022444.3	solute carrier family 13 member 1 (SLC13A1), transcript variant 1		6076	6518	7610	6120
NM_000165.4	Gap junction protein alpha 1 (GJA1)		5631	5946	7240	6464
NM_000339.2	solute carrier family 12 member 3 (SLC12A3), transcript variant 1		5474	5598	7408	5521
NM_001099660.1	leucine rich repeat neuronal 3 (LRRN3), transcript variant 1		4671	7080	5760	5680
NM_020774.3	mindbomb E3 ubiquitin protein ligase 1 (MIB1)		2446	2137	3173	2283
NM_001185072.2	Claudin 12 (CLDN12), transcript variant 1		1852	2011	2243	2018
NM_004093.3	Ephrin B2 (EFNB2)		1530	1246	3029	1666
NM_001897.4	chondroitin sulfate proteoglycan 4 (CSPG4)		1053	1159	3238	2230
NM_005602.5	Claudin 11 (CLDN11), transcript variant 1		654	660	1022	765
NM_001004431.2	meteorin like, glial cell differentiation regulator (METRNL)		576	835	933	649
NM_001004356.2	fibroblast growth factor receptor-like 1 (FGFRL1), transcript variant 1		559	602	1312	884
NM_004172.4	Solute carrier family 1 member 3 (SLC1A3), transcript variant GLAST		431	466	573	409
NM_001005388.2	neurofascin (NFASC), transcript variant 1		322	357	659	441
NM_020455.5	adhesion G protein-coupled receptor G6 (ADGRG6), transcript variant a1		320	223	360	226
NM_198569.2	adhesion G protein-coupled receptor G6 (ADGRG6), transcript variant b1		317	220	356	225
NM_006617.1	nestin (NES)		313	252	559	329
NM_023004.5	reticulon 4 receptor (RTN4R)		153	158	272	181
NM_002055.4	glial fibrillary acidic protein (GFAP), transcript variant 1		149	123	197	98
NM_018647.3	TNF receptor superfamily member 19 (TNFRSF19), transcript variant 1		122	91	158	83
NM_001302688.1	Apolipoprotein E (APOE), transcript variant 1		97	81	109	69

NM_006206.4	platelet derived growth factor receptor alpha (PDGFRA)		83	74	126	82
NM_000067.2	Carbonic anhydrase 2 (CA2), transcript variant 1		83	73	106	65
NM_000141.4	fibroblast growth factor receptor 2 (FGFR2), transcript variant 1		74	68	148	86
NM_002442.3	musashi RNA binding protein 1 (MSI1)		61	49	143	95
NM_005806.3	oligodendrocyte lineage transcription factor 2 (OLIG2)		50	72	92	55
NM_002011.4	fibroblast growth factor receptor 4 (FGFR4), transcript variant 1		41	49	73	53
NM_003057.2	solute carrier family 22 member 1 (SLC22A1), transcript variant 1		37	57	53	46
NM_001301186.1	leucine rich repeat and Ig domain containing 1 (LINGO1), transcript variant 2		30	28	73	33
NM_032808.6	leucine rich repeat and Ig domain containing 1 (LINGO1), transcript variant 1		30	28	73	33
NM_006574.3	chondroitin sulfate proteoglycan 5 (CSPG5), transcript variant 1		29	40	61	35
NM_001206943.1	chondroitin sulfate proteoglycan 5 (CSPG5), transcript variant 3		29	40	61	36
NM_000142.4	fibroblast growth factor receptor 3 (FGFR3), transcript variant 1		26	35	42	35
NM_001163213.1	fibroblast growth factor receptor 3 (FGFR3), transcript variant 3		25	34	42	35
NM_022965.3	fibroblast growth factor receptor 3 (FGFR3), transcript variant 2		24	33	40	34
NM_006941.3	SRY-box 10 (SOX10)		23	22	54	48
NM_138983.2	oligodendrocyte transcription factor 1 (OLIG1)		23	19	39	31
NM_002507.3	nerve growth factor receptor (NGFR)		20	10	31	20
NM_002544.4	oligodendrocyte myelin glycoprotein (OMG)		15	14	13	14
NM_006271.1	S100 calcium binding protein A1 (S100A1)		15	13	20	11
NM_000533.4	proteolipid protein 1 (PLP1), transcript variant 1		13	11	20	9
NM_001128310.2	SPARC like 1 (SPARCL1), transcript variant 1		13	12	14	10
NM_001315491.1	myelin protein zero (MPZ), transcript variant 1		12	10	19	16
NM_001324188.1	leucine rich repeat neuronal 1 (LRRN1), transcript variant 1		10	8	13	7
NM_001025101.1	myelin basic protein (MBP), transcript variant 7		10	8	10	8
NM_178568.3	reticulon 4 receptor like 1 (RTN4RL1)		7	8	16	9
NM_001025081.1	myelin basic protein (MBP), transcript variant 1		7	6	8	6
NM_022829.5	solute carrier family 13 member 3 (SLC13A3), transcript variant 1		6	5	13	8
NM_001101391.1	leucine rich repeat and Ig domain containing 3 (LINGO3)		5	4	6	2
NM_152570.2	leucine rich repeat and Ig domain containing 2 (LINGO2), transcript variant 1		2	3	5	3

NM_175747.2	oligodendrocyte transcription factor 3 (OLIG3)		2	2	3	3
NM_006272.2	S100 calcium binding protein B (S100B)		0	0	2	0
NM_002361.3	myelin associated glycoprotein (MAG), transcript variant 1		0	0	0	0
NM_080600.2	myelin associated glycoprotein (MAG), transcript variant 2		0	0	0	0
NM_001199216.1	myelin associated glycoprotein (MAG), transcript variant 3		0	0	0	0

Table S2: List of selected neuronal cell markers and number of mapped reads in DAOY cells. Each value represents an average number of reads from three independent biological replicates used for transcriptome analysis.

accession number	sample ID	TBEV	+	+	-	-
		IFN-β	+	-	+	-
NM_006086.3	tubulin beta 3 class III (TUBB3), transcript variant 1		16923	16705	24429	16488
NM_001197181.1	tubulin beta 3 class III (TUBB3), transcript variant 2		16858	16645	24330	16421
NM_001080497.2	multiple EGF like domains 9 (MEGF9)		13899	14961	18924	14712
NM_006288.4	Thy-1 cell surface antigen (THY1), transcript variant 1		4838	4456	7260	4637
NM_001311160.1	Thy-1 cell surface antigen (THY1), transcript variant 2		4808	4425	7209	4616
NM_001311162.1	Thy-1 cell surface antigen (THY1), transcript variant 3		4799	4418	7198	4607
NM_006158.4	neurofilament, light polypeptide (NEFL)		4500	3528	6245	3192
NM_015331.2	nicastrin (NCSTN), transcript variant 1		4461	4080	6857	4347
NM_001077628.2	aph-1 homolog A, gamma-secretase subunit (APH1A), transcript variant 1		4318	4397	5194	4243
NM_006516.2	solute carrier family 2 member 1 (SLC2A1)		4135	4367	7736	5352
NM_000021.3	presenilin 1 (PSEN1), transcript variant 1		3633	3772	4722	3445
NM_005638.5	vesicle associated membrane protein 7 (VAMP7), transcript variant 1		2367	2263	2831	2197
NM_001159999.2	neuregulin 1 (NRG1), transcript variant HRG-beta1b		2165	2275	3306	2180
NM_172341.2	presenilin enhancer gamma-secretase subunit (PSENEN), transcript variant 1		2132	2125	2240	1847
NM_004603.3	syntaxin 1A (STX1A), transcript variant 1		1891	2299	2775	2272
NM_005078.3	transducin like enhancer of split 3 (TLE3), transcript variant 1		1392	1400	3506	2170
NM_002856.2	nectin cell adhesion molecule 2 (NECTIN2), transcript variant alpha		844	1018	1517	906
NM_001042724.1	nectin cell adhesion molecule 2 (NECTIN2), transcript variant delta		789	932	1497	895
NM_001278116.1	L1 cell adhesion molecule (L1CAM), transcript variant 4		785	925	1943	1245
NM_001146054.1	synuclein alpha (SNCA), transcript variant 2		647	616	758	513
NM_001145110.1	neural EGFL like 2 (NELL2), transcript variant 5		457	394	585	407
NM_001321074.1	discs large MAGUK scaffold protein 4 (DLG4), transcript variant 3		384	379	629	421
NM_003761.4	vesicle associated membrane protein 8 (VAMP8)		345	323	413	267
NM_014232.2	vesicle associated membrane protein 2 (VAMP2), transcript variant 1		263	265	453	305
NM_022370.3	roundabout guidance receptor 3 (ROBO3)		253	318	358	278
NM_014231.4	vesicle associated membrane protein 1 (VAMP1), transcript variant 1		239	220	392	229
NM_006634.2	vesicle associated membrane protein 5 (VAMP5)		233	210	218	174

NM_003179.2	synaptophysin (SYP)		229	229	340	224
NM_000817.2	glutamate decarboxylase 1 (GAD1), transcript variant GAD67		103	84	127	78
NM_001123066.3	microtubule associated protein tau (MAPT), transcript variant 6		82	70	133	90
NM_013445.3	glutamate decarboxylase 1 (GAD1), transcript variant GAD25		67	57	72	56
NM_032727.3	internexin neuronal intermediate filament protein alpha (INA)		61	92	116	108
NM_014839.4	phospholipid phosphatase related 4 (PLPPR4), transcript variant 1		55	38	78	44
NM_005382.2	neurofilament, medium polypeptide (NEFM), transcript variant 1		45	46	68	42
NM_002374.3	microtubule associated protein 2 (MAP2), transcript variant 1		37	28	38	20
NM_031847.2	microtubule associated protein 2 (MAP2), transcript variant 4		34	27	35	17
NM_007327.3	glutamate ionotropic receptor NMDA type subunit 1 (GRIN1), transcript variant GluN1-1a		28	20	38	20
NM_001185090.1	glutamate ionotropic receptor NMDA type subunit 1 (GRIN1), transcript variant GluN1-3b		25	17	34	17
NM_001105541.1	neurofilament, medium polypeptide (NEFM), transcript variant 2		11	11	24	10
NM_021076.3	neurofilament heavy polypeptide (NEFH)		9	28	21	15
NM_022363.2	LIM homeobox 5 (LHX5)		8	10	18	9
NM_001135659.2	neurexin 1 (NRXN1), transcript variant alpha2		3	5	6	3
NM_002500.4	neuronal differentiation 1 (NEUROD1)		2	2	3	2
NM_006160.3	neuronal differentiation 2 (NEUROD2)		2	1	3	1
NM_199292.2	tyrosine hydroxylase (TH), transcript variant 1		1	1	4	1
NM_001288713.1	neural EGFL like 1 (NELL1), transcript variant 3		0	0	0	0
NM_000818.2	glutamate decarboxylase 2 (GAD2), transcript variant 1		0	1	0	0
NM_001134366.1	glutamate decarboxylase 2 (GAD2), transcript variant 2		0	1	0	0

Table S3: List of genes found to be differentially expressed (q>0.05) using Cuffdiff in DAOY cells. All three comparative datasets are included (column 1: TBEV-infected; column 2: IFN-β-treated; column 3: IFN-β-pretreated + TBEV-infected).

TBEV		
name	log2 change	q-value
IFNL1	7,45481	0,00213657
CXCL11	4,91223	0,00213657
CXCL10	4,84894	0,00213657
CCL4L2	4,07903	0,0327467
IFIT2	3,76447	0,00213657
TAC3	3,52009	0,00213657
SPRR2D	3,46445	0,00213657
CCL5	3,42367	0,00213657
RANBP3L	3,1796	0,00213657
IFIT1	3,1723	0,00213657
RAET1L	3,08861	0,00213657
RSAD2	2,84629	0,00213657
H19	2,81375	0,00213657
OASL	2,79084	0,00213657
KRT16	2,67213	0,00213657
TAC1	2,52424	0,00213657
KRT14	2,49443	0,00213657
SCN3A	2,39884	0,00213657
RP11-757G1.6	2,34956	0,00213657
DDIT3	2,26698	0,00213657
IFIT3	2,24483	0,00213657
KCNV1	2,23128	0,00213657
KRT17	2,15607	0,0466334
KLF4	2,09178	0,00213657
IFIH1	2,01993	0,00213657
HERPUD1	2,01607	0,00213657
RP11-495P10.9	1,97995	0,00213657
TRIB3	1,96088	0,00213657
FAM65B	1,95611	0,00213657
IL18RAP	1,94368	0,045023
GBP5	1,91198	0,00213657
OAS2	1,90905	0,00213657
CCL4	1,90677	0,00213657
HSPA5	1,87058	0,00213657
LRMP	1,86646	0,00875918
SDF2L1	1,83852	0,00213657
RP11-495P10.8	1,83453	0,0345094
EPSTI1	1,83262	0,00213657
ATF3	1,8221	0,00213657
ACTN2	1,80091	0,00213657
INHBE	1,78536	0,00213657
HIP1R	1,78173	0,00213657

IFN-β		
name	log2 change	q-value
IFI27	5,02376	0,00772276
IFI6	4,21077	0,00772276
RNA5-8SP6	3,65326	0,00772276
RMRP	3,43593	0,00772276
RN7SK	3,26718	0,00772276
IFITM1	3,20318	0,00772276
AC010970.2	2,81071	0,00772276
BST2	1,92617	0,00772276
EPSTI1	1,44392	0,00772276
RP5-1021I20.2	1,41367	0,00772276
AC187652.1	1,26838	0,00772276
RUNX1T1	1,17258	0,00772276
CRABP2	1,17087	0,00772276
RP11-260A9.6	1,14568	0,00772276
IFITM3	1,0781	0,00772276
ST8SIA6	1,0526	0,0187021
RGCC	1,05189	0,00772276
PADI3	1,03566	0,00772276
SERPING1	1,01323	0,0375334
CT45A5	0,989782	0,0240663
LL22NC03-2H8.4	0,962085	0,028587
KLF8	0,95688	0,00772276
HSP90AB3P	0,951043	0,00772276
RP11-389K14.3	0,92672	0,0187021
IFI44L	0,9221	0,00772276
PALM3	0,910223	0,028587
HAPLN1	0,895136	0,028587
MX1	0,894832	0,00772276
FILIP1L	0,878775	0,0240663
HLA-B	0,869312	0,00772276
RNASE1	0,862023	0,00772276
CFH	0,856972	0,00772276
TSHZ2	0,842123	0,014176
TNFSF18	0,839424	0,028587
XAF1	0,835209	0,00772276
PRR15	0,833425	0,0187021
NKX2-8	0,82798	0,00772276
ZNF608	0,826708	0,00772276
PBX1	0,82292	0,00772276
ISG15	0,821807	0,00772276
EEF1A1P11	0,816966	0,00772276
MARC1	0,801411	0,0333823

IFN-β + TBEV		
Name	log2 change	q-value
IFI27	5,48133	0,00148451
IFI6	4,74221	0,00148451
IFITM1	3,67316	0,00148451
BST2	2,34874	0,00148451
RMRP	1,91371	0,00148451
EPSTI1	1,81611	0,00148451
KCNIP1	1,81115	0,00148451
HPGD	1,70715	0,0160813
CTD-2566J3.1	1,66643	0,044652
GALNT15	1,6245	0,00148451
RP5-1021I20.2	1,58356	0,00148451
RN7SK	1,52879	0,00148451
IFITM3	1,39804	0,00148451
CRABP2	1,37193	0,00148451
STMN2	1,36186	0,011309
PCDP1	1,35395	0,014489
ISG15	1,29774	0,00148451
RGCC	1,2973	0,00148451
TNFSF18	1,25089	0,00148451
RNA5-8SP6	1,23803	0,00148451
XXbac-BPG248L24.12	1,21027	0,00776792
HLA-B	1,18276	0,00148451
RSAD2	1,18247	0,00148451
RP11-260A9.6	1,15924	0,00267331
SERPING1	1,15197	0,00148451
RGS1	1,13588	0,00148451
HAPLN1	1,12568	0,00148451
IFI44L	1,12107	0,00148451
RUNX1T1	1,11186	0,00148451
NXNL2	1,09386	0,0275868
CFH	1,08059	0,00148451
IFITM2	1,06688	0,00148451
GLTPD2	1,05294	0,0452196
RP4-794H19.4	1,03974	0,0423086
FILIP1L	1,02952	0,00148451
MORN3	1,02668	0,0269749
RP11-389K14.3	1,01977	0,00148451
RBP1	1,01295	0,00148451
DMRT1	1,00895	0,0136673
KRT14	0,997822	0,00148451
GARNL3	0,986897	0,00583817
PKIB	0,958106	0,00148451

GDF15	1,77547	0,00213657
ZBTB32	1,72271	0,00213657
DDX58	1,67267	0,00213657
GBP4	1,63622	0,00213657
SESN2	1,63081	0,00213657
BBC3	1,62769	0,00213657
RP11-42O15.3	1,61101	0,00213657
RTP4	1,60973	0,00213657
CHAC1	1,59871	0,00213657
ISG15	1,59324	0,00213657
DHX58	1,55253	0,00213657
HERC5	1,54041	0,00213657
OTUD1	1,5328	0,00213657
TG	1,52259	0,0442766
ERO1LB	1,51163	0,00213657
PRR15	1,5088	0,00213657
SECTM1	1,50377	0,0130136
ISG20	1,50163	0,00213657
IL12A	1,49528	0,00213657
TNF	1,48774	0,00213657
RARRES3	1,47668	0,00213657
CCL3L1	1,46666	0,025121
PMAIP1	1,4445	0,00213657
MANF	1,43436	0,00213657
CEACAM1	1,4035	0,0167771
RP11-442H21.2	1,39982	0,00213657
AC069363.1	1,34959	0,00213657
NEURL3	1,34321	0,0345094
STK32A	1,33977	0,0142472
PTGER4	1,33113	0,00213657
ALDH1L2	1,32915	0,00213657
MISP	1,31799	0,00213657
IFI27	1,3128	0,00213657
CTH	1,31016	0,00213657
DNAJB9	1,2981	0,00213657
SP8	1,28585	0,00213657
RUNX1T1	1,28116	0,00213657
ADAP1	1,28011	0,0345094
DDIT4	1,27938	0,00213657
VIPR2	1,27435	0,00213657
RND1	1,25219	0,00213657
SAMD9	1,24145	0,00213657
FST	1,2365	0,00875918
STMN2	1,22761	0,028669
XBP1	1,22503	0,00213657

HDGFP1	0,790177	0,0187021
RP11-764K9.4	0,770225	0,00772276
LL22NC03-2H8.5	0,757043	0,00772276
PALMD	0,732732	0,00772276
ERVWE2	0,70601	0,00772276
MX2	0,703355	0,00772276
ESX1	0,693078	0,00772276
FRAS1	0,688424	0,00772276
HMGN5	0,686933	0,00772276
EFEMP1	0,682309	0,00772276
RP5-1172A22.1	0,675371	0,028587
DMBX1	0,672387	0,0422388
MXRA5	0,664725	0,0375334
ADAMTS15	0,662422	0,014176
MEST	0,662209	0,00772276
MDFI	0,650275	0,00772276
AFAP1L2	0,639936	0,00772276
GMPR	0,618991	0,00772276
RP11-51M18.1	0,603053	0,0187021
IGFBP5	0,600861	0,0187021
CTA-392C11.1	0,587778	0,0187021
PKIB	0,587209	0,0470386
POSTN	0,575424	0,0187021
DSP	0,57019	0,00772276
MNS1	0,562102	0,0240663
RP11-946L20.4	0,562094	0,028587
LY6E	0,560834	0,00772276
PTN	0,543484	0,014176
AHNAK	0,526288	0,014176
DCLK2	0,522099	0,0240663
HLA-C	0,509553	0,00772276
B2M	0,50688	0,014176
LIMA1	0,489256	0,0187021
NEDD9	0,461945	0,00772276
IGFBP3	0,457224	0,0187021
MRPL41	-0,446487	0,0333823
RPL39	-0,449443	0,028587
RPL34	-0,453642	0,0187021
SLC39A14	-0,458045	0,014176
GAL	-0,459702	0,0375334
AKR1B1	-0,467272	0,00772276
ATP6V0B	-0,468365	0,014176
SLC2A6	-0,472253	0,0375334
TFAP2C	-0,489706	0,0333823
NDUFB1	-0,491573	0,0240663

RP11-449H11.1	0,955224	0,0339714
PPP1R1C	0,95462	0,044652
PADI3	0,952332	0,00148451
HLA-DRA	0,948934	0,0153371
PPP1R14A	0,942616	0,0492892
SEMA3E	0,925568	0,0492892
CFD	0,910523	0,00148451
MX1	0,904788	0,00148451
XAF1	0,89354	0,00148451
SOCS2	0,882928	0,00148451
RNASE1	0,880832	0,00148451
HYAL4	0,876708	0,0153371
MTUS1	0,871939	0,011309
PTN	0,862004	0,00148451
HNMT	0,859566	0,00776792
CT45A5	0,857044	0,0207619
PARK2	0,850136	0,00148451
CHAC1	0,836144	0,00148451
CT49	0,832496	0,0128531
RP5-1172A22.1	0,831472	0,00148451
C8orf47	0,821881	0,00148451
KLF8	0,814771	0,00148451
PALMD	0,811232	0,00148451
TEX40	0,799009	0,0120659
SLC16A14	0,791881	0,00267331
B2M	0,787633	0,00148451
CDC42EP5	0,786816	0,0429875
CTD-2147F2.1	0,78011	0,0243368
CHRNA9	0,769848	0,00148451
HSP90AB3P	0,769692	0,0153371
GGACT	0,767183	0,044652
ANXA3	0,765887	0,00267331
APOC1	0,752203	0,0048506
RARRES2	0,750536	0,00148451
CFI	0,749691	0,00148451
RARRES3	0,747992	0,00148451
BIRC7	0,744607	0,00680068
RGS2	0,735428	0,00148451
NKX2-8	0,735055	0,00148451
CXCL10	0,734649	0,00583817
RP11-398E10.1	0,729071	0,00148451
RASSF10	0,723432	0,00776792
EFEMP1	0,719187	0,00148451
LINC00607	0,717437	0,00776792
TSPAN2	0,716559	0,00148451

RP11-475I24.8	1,21943	0,00213657
CCL3	1,20625	0,00213657
RP11-109M17.2	1,20423	0,0442766
TNFSF18	1,20146	0,00213657
ADM2	1,18749	0,00213657
ASNSP1	1,18413	0,0355607
MAL2	1,15645	0,00213657
DNAJC3	1,15281	0,00213657
PIWIL1	1,14939	0,00213657
FKBP9L	1,13981	0,00213657
IDO1	1,13962	0,00213657
LTA	1,13573	0,0274911
PLCG2	1,12739	0,00213657
NUPR1	1,10733	0,00213657
SEL1L	1,09617	0,00213657
PLEKHA4	1,09375	0,00213657
RXRG	1,09082	0,0130136
RAB39B	1,08922	0,00213657
AIM2	1,08597	0,00213657
PIK3AP1	1,08319	0,00213657
TRAF1	1,07879	0,00213657
CRELD2	1,06479	0,00213657
FAM129A	1,05358	0,00213657
NUCB2	1,04853	0,00213657
CD69	1,0456	0,00213657
SNPH	1,04535	0,00213657
RGS16	1,04199	0,00213657
DNAJC3-AS1	1,03114	0,00397639
ST8SIA4	1,02251	0,025121
FGF2	1,0136	0,00213657
SEMA3D	1,0066	0,00213657
WARS	1,00616	0,00213657
HES4	1,0021	0,00213657
NOL4	0,999912	0,0409
ASNS	0,997397	0,00213657
AKNA	0,979182	0,00213657
DNAJB11	0,977298	0,00213657
EPHA4	0,968176	0,0072481
MCF2L	0,966103	0,00213657
SLC7A11	0,961583	0,00213657
TMEM47	0,956618	0,00213657
HERC6	0,956438	0,00213657
ABCG1	0,949994	0,00213657
LURAP1L	0,945858	0,00213657
ALPK2	0,943142	0,00875918

UQCRQ	-0,497001	0,00772276
KIAA1462	-0,49979	0,00772276
TSPO	-0,501563	0,014176
RAB3B	-0,50486	0,014176
TMEM208	-0,509336	0,00772276
PLAU	-0,515178	0,00772276
ACO1	-0,516127	0,00772276
IFI27L2	-0,518031	0,00772276
DUSP1	-0,51998	0,00772276
IRAK2	-0,520086	0,00772276
PTGES	-0,525814	0,0375334
RPS21	-0,532757	0,00772276
C12orf57	-0,546182	0,00772276
PREX1	-0,549901	0,00772276
ZNF469	-0,557916	0,0187021
PTPRN2	-0,558316	0,00772276
PPAP2B	-0,562734	0,00772276
CDKN1A	-0,566475	0,00772276
SGPP2	-0,572207	0,0187021
ICOSLG	-0,572947	0,014176
LINC00622	-0,592406	0,00772276
MT1F	-0,603233	0,0187021
CORO2B	-0,604857	0,014176
MIR146A	-0,615279	0,00772276
LAPTM5	-0,617529	0,00772276
CLDN1	-0,618819	0,0187021
MPP4	-0,623089	0,0187021
TMEM159	-0,624358	0,0187021
TSC22D1	-0,629162	0,00772276
RPS29	-0,629399	0,00772276
SERPINE1	-0,631488	0,00772276
AKR1C3	-0,644518	0,0422388
TNFSF9	-0,646763	0,00772276
RNF144B	-0,659725	0,0240663
SIGLEC15	-0,672794	0,00772276
LUM	-0,686504	0,00772276
EEF1A1P13	-0,695756	0,00772276
TFPI2	-0,69747	0,00772276
TMEM255B	-0,699088	0,0375334
HBEGF	-0,69951	0,00772276
MAFB	-0,709805	0,0187021
C1QTNF1	-0,714073	0,00772276
ADAM8	-0,734325	0,00772276
PLAUR	-0,740149	0,00772276
GDF15	-0,740742	0,0470386

LY6E	0,714593	0,00148451
PBX1	0,714411	0,00148451
IGFBP3	0,705104	0,00148451
MDFI	0,702009	0,00148451
SGCD	0,697118	0,00381732
C6orf226	0,694152	0,00776792
RP4-555D20.4	0,692359	0,0487479
HIST1H2AC	0,689531	0,00148451
RP11-865I6.2	0,687772	0,00148451
ARHGAP36	0,686791	0,0160813
LINC01003	0,683992	0,00148451
GMPR	0,678787	0,00148451
CMPK2	0,672101	0,0275868
ADAMTS16	0,670336	0,00381732
PARP9	0,669614	0,0105503
CCDC34	0,666117	0,00148451
CXorf22	0,664854	0,0333646
POSTN	0,66445	0,00148451
HMGN5	0,664265	0,00148451
CNTNAP4	0,66026	0,0105503
GLUD2	0,659446	0,00381732
HLA-F	0,654519	0,00148451
ZNF608	0,649268	0,0048506
SESN3	0,647166	0,00583817
MTND4P12	0,647028	0,00148451
RAB39B	0,646396	0,00148451
C11orf70	0,645074	0,00965725
RP11-757G1.6	0,642574	0,0257637
RASSF9	0,636344	0,00148451
GNG2	0,636026	0,00148451
HIST3H2A	0,635582	0,00148451
DMKN	0,631316	0,00148451
MGP	0,630825	0,0459156
CTA-392C11.1	0,627831	0,00148451
STON1	0,625004	0,00148451
HLA-C	0,618047	0,00148451
HIST1H2BO	0,615614	0,0184004
TMEM160	0,606608	0,00148451
PLAC8	0,60284	0,0048506
AIM2	0,602629	0,00148451
PTMAP5	0,601945	0,00267331
CRIP1	0,590964	0,0120659
RP11-111M22.3	0,588684	0,00148451
MDK	0,585695	0,00148451
BMP5	0,585052	0,0315796

IL6	0,933491	0,00213657
HSP90B1	0,930262	0,00213657
SLC6A9	0,922219	0,00213657
MTHFD2	0,919512	0,00213657
MX2	0,914548	0,00213657
JDP2	0,910709	0,00213657
RP11-260A9.6	0,904454	0,025121
GALNT3	0,902987	0,00213657
ZC3HAV1	0,886006	0,00213657
PCK2	0,854998	0,0072481
CA3	0,854117	0,00213657
RCAN1	0,849171	0,00213657
BCYRN1	0,846528	0,00213657
IFI6	0,837502	0,00213657
MMP13	0,835199	0,025121
PSAT1	0,833606	0,00213657
EVA1C	0,830829	0,0167771
NOV	0,828102	0,00213657
PDIA4	0,821628	0,00213657
PAK3	0,812663	0,0419519
PPP1R15A	0,8068	0,00213657
XAF1	0,805379	0,00213657
DDX60	0,802803	0,00213657
CBS	0,801146	0,00213657
SLFN11	0,800772	0,00213657
JAK2	0,797172	0,00213657
CT45A5	0,796905	0,0496511
FAM46A	0,792592	0,00213657
RP11-1151B14.4	0,792551	0,00397639
LARP1B	0,788852	0,00213657
TNFRSF9	0,78683	0,00213657
CTD-2227E11.1	0,782574	0,0056613
IGFBP6	0,779283	0,00213657
GTF2H2B	0,77913	0,0072481
MXD1	0,771907	0,0218788
GFPT1	0,770271	0,0401306
RHEBL1	0,767644	0,00213657
MX1	0,765353	0,00213657
EDN1	0,762495	0,00213657
HYOU1	0,761924	0,00213657
SYT1	0,752924	0,0056613
BST2	0,75268	0,00213657
RP11-597K23.2	0,723153	0,0142472
PPM1K	0,722459	0,00213657
CLDN1	0,720606	0,00213657

CTD-2328D6.1	-0,758721	0,0375334
PIM2	-0,765002	0,00772276
CSF2	-0,77959	0,00772276
LIF	-0,780291	0,00772276
C8orf4	-0,782414	0,00772276
HMOX1	-0,799996	0,00772276
GPR68	-0,811991	0,00772276
SFRP2	-0,814766	0,00772276
CACNA2D4	-0,835636	0,028587
LRRC55	-0,851095	0,00772276
ABCA1	-0,85626	0,00772276
DDIT4	-0,860534	0,00772276
SH2D2A	-0,863496	0,00772276
ROBO4	-0,874679	0,0187021
KYNU	-0,882586	0,00772276
C2CD4A	-0,937151	0,00772276
SCG5	-0,952472	0,00772276
C11orf96	-0,988496	0,00772276
ASB2	-1,00633	0,00772276
AC003092.1	-1,01528	0,00772276
G0S2	-1,02286	0,00772276
MTMR9LP	-1,03027	0,00772276
CDX1	-1,03864	0,00772276
RAB27B	-1,04085	0,00772276
SPP1	-1,04829	0,00772276
RN7SL2	-1,05063	0,00772276
STC1	-1,09877	0,00772276
RP11-408P14.1	-1,10151	0,0422388
LINC00520	-1,105	0,00772276
CCL20	-1,10502	0,00772276
LUCAT1,RP11-213H15.4	-1,1242	0,0333823
AMPD3	-1,12558	0,00772276
CXCL3	-1,13769	0,00772276
RP11-211G23.2	-1,1391	0,0187021
C15orf48	-1,1434	0,00772276
IL6	-1,16583	0,00772276
MMP1	-1,17025	0,00772276
C4orf26	-1,18327	0,00772276
SCARA5	-1,19024	0,014176
CXCL1	-1,23935	0,00772276
CXCL2	-1,25673	0,00772276
RP11-443P15.2	-1,26862	0,0333823
PAX8-AS1	-1,29118	0,00772276
AKR1C1	-1,30151	0,00772276
RP11-907D1.2	-1,3079	0,00772276

HES4	0,584334	0,0136673
CYP4X1	0,582897	0,0120659
MX2	0,582299	0,00381732
ZMAT4	0,582099	0,0105503
EEF1A1P11	0,577417	0,00267331
PLK2	0,573702	0,00680068
SUGCT	0,571044	0,00148451
OR7E38P	0,569475	0,00148451
RP11-764K9.4	0,560043	0,00148451
LINC01139	0,552951	0,00381732
ALX1	0,547454	0,011309
LRRCC1	0,54403	0,00148451
SPATA17	0,537256	0,00680068
FAM133A	0,535453	0,00148451
BCYRN1	0,532118	0,00148451
GLT8D2	0,530745	0,00583817
CCDC89	0,528946	0,0288277
LAMA4	0,528365	0,0344907
CTAG2	0,527104	0,0423086
PPARG	0,524002	0,00148451
RP6-65G23.3	0,522736	0,0160813
SPANXD	0,521628	0,00776792
TFDP2	0,518867	0,00965725
LIMA1	0,518026	0,00148451
MEST	0,517563	0,00267331
PLBD1	0,516238	0,0275868
AC125232.1	0,51582	0,0320797
S100A10	0,515603	0,00148451
RPL41	0,513364	0,00148451
HIST1H2BD	0,511338	0,00148451
CCL5	0,507637	0,0048506
STK38L	0,505391	0,0215294
AOAH	0,50492	0,0368232
MNS1	0,504448	0,00148451
IDNK	0,502081	0,0327252
TPGS1	0,500881	0,00680068
ACTBL2	0,49998	0,00267331
LINC00460	0,499809	0,0435682
IFIH1	0,498112	0,00148451
ACSS3	0,497189	0,00148451
BEX1	0,49686	0,00148451
CD24P4	0,49667	0,00148451
PRKCDBP	0,494398	0,00148451
NKX2-1	0,49396	0,0243368
HHAT	0,491912	0,00148451

CITED2	0,71706	0,00213657
PARP14	0,715474	0,00213657
CD274	0,712463	0,00875918
SFN	0,705911	0,0142472
KIAA0040	0,698287	0,00213657
HKDC1	0,69752	0,0218788
RP11-37B2.1	0,695889	0,0167771
ANKRD33B	0,689546	0,0466334
SAMD9L	0,686037	0,00213657
APOC1	0,677418	0,0355607
GARS	0,67331	0,00213657
PELI1	0,669782	0,00213657
NCOA7	0,667172	0,00213657
FKBP2	0,666167	0,00213657
CXCL2	0,661801	0,00213657
GLUD2	0,661349	0,0056613
IL23A	0,660318	0,022904
IRF1	0,654407	0,0072481
PRSS16	0,651232	0,00213657
CFD	0,643759	0,0466334
SELK	0,641441	0,00213657
PPIB	0,636141	0,00213657
DNAJC12	0,634439	0,00213657
DNAJB5	0,631603	0,00213657
RSBN1L-AS1	0,628898	0,00213657
PARM1	0,62363	0,0485774
EFNA1	0,62314	0,00213657
IL15	0,622579	0,0142472
EIF2AK3	0,621269	0,0218788
IFIT5	0,615865	0,00213657
SLC33A1	0,609133	0,00213657
SARS	0,605478	0,00213657
FAM107B	0,605382	0,00213657
PPP4R4	0,605112	0,00213657
SDC4	0,602629	0,00213657
DERL2	0,601104	0,00875918
SEC24D	0,59794	0,00213657
RHOB	0,5976	0,00213657
BIRC3	0,597455	0,00213657
UBE2J1	0,591329	0,00213657
RIPK2	0,58949	0,00213657
USP18	0,586759	0,00213657
NFKBIZ	0,586436	0,0167771
APOL6	0,584814	0,00213657
CEBPG	0,583733	0,00213657

CTD-2319I12.1	-1,31206	0,00772276
IL1A	-1,34978	0,00772276
RPL24P2	-1,36203	0,00772276
PTGS2	-1,38772	0,00772276
BCL2A1	-1,39363	0,00772276
CLDN14	-1,40441	0,00772276
IL1RN	-1,43968	0,00772276
CXCL5	-1,44071	0,00772276
IL11	-1,47491	0,00772276
SERPINB2	-1,49855	0,00772276
ESM1	-1,66775	0,00772276
IL8	-1,69261	0,00772276
SLCO2B1	-1,69826	0,00772276
HCK	-1,75009	0,00772276
SLAMF8	-1,86425	0,00772276
ITGAX	-1,91489	0,028587
IL1B	-2,1976	0,00772276
CSF3	-2,38053	0,00772276
CCL3	-2,6512	0,00772276
IL36RN	-3,02785	0,00772276

PRSS23	0,491293	0,00680068
TCEAL2	0,491282	0,00148451
RPL35	0,488882	0,00148451
RP3-512B11.3	0,486243	0,00965725
ESX1	0,485399	0,00680068
GADD45GIP1	0,481162	0,00148451
GSTK1	0,480234	0,00148451
TMEM121	0,479665	0,0350833
ARTN	0,478393	0,0387115
HIST1H1C	0,47743	0,00148451
IFI35	0,476863	0,011309
TNFRSF19	0,475395	0,00965725
ZNF441	0,473228	0,0477563
SDC2	0,473101	0,0288277
NEFL	0,470056	0,00148451
LGR4	0,467969	0,00381732
NNMT	0,465032	0,00267331
TPPP3	0,462312	0,0275868
TMEM134	0,461925	0,0275868
SLC25A18	0,46143	0,049862
CLGN	0,460927	0,00267331
RP4-665J23.1	0,460715	0,0128531
OASL	0,460111	0,00267331
S100A2	0,458791	0,00148451
TRAPPC5	0,458636	0,00148451
AC009518.4	0,457724	0,0282818
FAM195A	0,457579	0,00871429
RTP4	0,456928	0,0320797
C1orf53	0,455346	0,0264419
IFI16	0,455022	0,00267331
COQ2	0,454808	0,0168687
AFAP1L2	0,450269	0,00583817
SEPW1	0,449237	0,0153371
FMO3	0,449036	0,0199666
PAGE1	0,441237	0,0128531
GBP1	0,43985	0,00583817
SERPINB6	0,437293	0,00148451
THYN1	0,435264	0,00267331
SERPINB1	0,433447	0,0435682
SEMA3C	0,432887	0,0048506
ANXA1	0,429909	0,00583817
RP11-51M18.1	0,429734	0,0176579
ASNS	0,429685	0,00381732
SAMD9	0,4292	0,00148451
SEMA3A	0,428737	0,0487479

SLC1A4	0,583144	0,00213657
CRELD1	0,581941	0,022904
AARS	0,580665	0,00213657
ZNF165	0,580185	0,00213657
CALR	0,579494	0,00213657
EDEM1	0,579243	0,0239927
TSC22D3	0,576419	0,00213657
CH25H	0,573501	0,00213657
TSPYL2	0,571111	0,0056613
MIS12	0,569249	0,0373435
FNDC3A	0,568739	0,00213657
BHLHE40	0,563855	0,0072481
LZTFL1	0,555487	0,0056613
VLDLR	0,554911	0,00213657
AC125232.1	0,552238	0,0218788
IFI16	0,551624	0,00213657
PLA2G3	0,551298	0,00213657
ZFP36	0,547459	0,00213657
DDR2	0,546552	0,00213657
ARHGEF2	0,546471	0,00213657
PHGDH	0,546267	0,00213657
NR4A3	0,54149	0,0156083
SETX	0,540854	0,00213657
APOL2	0,53986	0,00397639
CCNA1	0,537989	0,0072481
SPAG9	0,535083	0,0393926
GBP1	0,530544	0,00213657
PIGA	0,52776	0,00875918
MAGT1	0,525857	0,00213657
IFI44	0,525673	0,00213657
MBNL2	0,52534	0,0316437
MCEE	0,524783	0,0262606
C2CD4A	0,522159	0,0180821
GADD45A	0,519678	0,00213657
RP11-111M22.3	0,518732	0,0072481
SGPP2	0,514241	0,00213657
IL8	0,513404	0,00213657
ATF4	0,506979	0,00213657
FAM26E	0,506695	0,00213657
GTPBP2	0,506492	0,0460184
MILR1	0,505939	0,0373435
AKAP17A	0,504168	0,00213657
PDIA6	0,503859	0,00213657
IBTK	0,503001	0,00213657
AVPI1	0,497322	0,00213657

HLA-A	0,427621	0,00148451
OLFML3	0,426493	0,0243368
RPL13	0,423832	0,0048506
VAMP8	0,423542	0,0221892
CHCHD5	0,422868	0,0191454
ZNF311	0,420515	0,0327252
C21orf119	0,420358	0,0309692
PSME1	0,417433	0,00871429
ECI1	0,416626	0,0229553
SCG2	0,413482	0,0236514
HIST1H2BK	0,413333	0,00148451
STMN1	0,410356	0,00680068
FKBP7	0,410201	0,0423086
DAZ1	0,40987	0,049862
NAP1L3	0,409535	0,0136673
PRTFDC1	0,407797	0,0392928
NET1	0,406446	0,0215294
INPP4B	0,403217	0,0472024
CSRP2	0,403131	0,0105503
ARHGAP18	0,40173	0,0168687
CYP2J2	0,397283	0,0275868
CAT	0,395497	0,0191454
ICT1	0,393869	0,0048506
TNFSF10	0,389235	0,0184004
RPS27	0,387573	0,00871429
HIST1H2BC	0,384158	0,0355872
CDKN3	0,384073	0,0120659
NEDD9	0,383823	0,0120659
PDE4B	0,3835	0,0315796
BASP1	0,381586	0,0048506
HMGB1	0,381215	0,0350833
SF3B5	0,381202	0,00965725
C9orf3	0,377671	0,0333646
CPA4	0,37739	0,0136673
MBIP	0,374029	0,0282818
PTTG1	0,373754	0,00776792
EDF1	0,373432	0,0128531
RPL32	0,373096	0,0105503
UBE2L6	0,371875	0,011309
GAS1	0,369545	0,0392928
BTF3	0,366626	0,0405302
COMMD6	0,366493	0,0333646
RGS16	0,366338	0,0302768
NMI	0,365164	0,0184004
ARPC3	0,362023	0,0472024

BTN3A3	0,495676	0,0460184
STX11	0,4942	0,0056613
PJA2	0,493919	0,00213657
C8orf4	0,492541	0,0056613
RP11-865I6.2	0,492097	0,00397639
ZNF311	0,490718	0,0156083
TNFSF10	0,489345	0,0056613
GBP2	0,489258	0,00397639
TRIM5	0,488446	0,022904
VCAM1	0,487242	0,00213657
HIAT1	0,486123	0,0056613
CREBRF	0,48214	0,0218788
SEC11C	0,481752	0,0307295
CDK2AP2	0,481253	0,00213657
PRKCDBP	0,48096	0,00213657
P2RX4	0,479914	0,0435674
IL1A	0,476815	0,00213657
PTN	0,474331	0,00213657
TRIM21	0,470545	0,0401306
BEX2	0,469225	0,0056613
PSPH	0,468577	0,0056613
LINC01003	0,467995	0,0142472
ZFAND2A	0,465834	0,0102076
PLEKHF1	0,46133	0,0180821
TMED7	0,460132	0,00397639
TDRD7	0,456468	0,00875918
CSF2	0,453233	0,00213657
MYC	0,453231	0,00397639
PYCR1	0,45066	0,0116711
CARS	0,450523	0,00213657
SAT1	0,447143	0,00213657
RPS6KC1	0,446229	0,0393926
ZMAT1	0,446038	0,0373435
BAMBI	0,443176	0,00213657
BTG2	0,442956	0,0307295
EEF1A1P11	0,442212	0,0339678
CORO1A	0,440785	0,0373435
SHMT2	0,440739	0,0142472
PDCD1LG2	0,436353	0,0142472
EAF2	0,434693	0,0373435
RPL41	0,434523	0,0072481
NEDD1	0,434107	0,0207525
VIMP	0,433642	0,0056613
GOLPH3L	0,431806	0,0142472
B3GNT2	0,41971	0,00875918

ACAA2	0,360591	0,0221892
PSME2	0,360405	0,0487479
HERC5	0,357703	0,0355872
RWDD2B	0,357197	0,0282818
EML4	0,355333	0,0302768
UQCRB	0,346905	0,0355872
C4orf27	0,346719	0,0465434
DBI	0,342423	0,0269749
TMEM14A	0,341814	0,0477563
PARP14	0,340562	0,0344907
HMGN1	0,336025	0,036194
CH25H	0,334198	0,044652
CENPW	0,332945	0,0399584
SNX7	0,331453	0,0387115
COX5B	0,331289	0,0243368
NEK2	0,327711	0,0483062
CCNB2	0,326853	0,0344907
CLEC11A	0,326647	0,0477563
LRRC40	0,32541	0,0374192
NDUFB7	0,324363	0,036194
AMTN	0,322666	0,0492892
RPS15	0,321174	0,0483062
SMARCA1	0,321039	0,0380385
EAPP	0,31871	0,044652
COPS4	0,313741	0,049862
MTCH1	-0,318207	0,0423086
ACLY	-0,319132	0,0477563
EIF4EBP2	-0,326868	0,0355872
ZNF697	-0,327254	0,0374192
MYBL2	-0,329582	0,0355872
UHRF1	-0,333191	0,0392928
ACVR1	-0,337542	0,0452196
PEX6	-0,34183	0,0320797
KDM3B	-0,347549	0,044652
CD81	-0,348417	0,0339714
VOPP1	-0,351001	0,0176579
SERPINE2	-0,352136	0,0320797
PCBP2	-0,355287	0,0392928
PFKL	-0,355353	0,0288277
PTPN1	-0,356484	0,0176579
ZNF318	-0,356596	0,0275868
WASL	-0,357373	0,0128531
INTS3	-0,358056	0,0236514
AKR1B1	-0,360253	0,0128531
EHD4	-0,361226	0,011309

LMO4	0,410931	0,0345094
ACSL3	0,405743	0,0072481
GBP3	0,402489	0,0316437
ISOC2	0,401825	0,0102076
ZNF804A	0,397925	0,0466334
ERP44	0,39146	0,00397639
EDF1	0,389939	0,0056613
BTG1	0,389619	0,0297104
TRAM1	0,388738	0,0435674
STX5	0,387429	0,0316437
PTX3	0,380209	0,0466334
PXK	0,379243	0,0345094
EMC2	0,378091	0,0297104
SPRY2	0,374452	0,0409
OSTC	0,372546	0,00875918
SLC7A5	0,372479	0,0167771
NFIL3	0,367508	0,045023
PRDX5	0,365784	0,0207525
GYPC	0,362557	0,0327467
LRRN3	0,362479	0,0239927
BET1	0,359301	0,0409
CETN2	0,358344	0,0442766
EIF4EBP1	0,357213	0,0419519
B2M	0,354041	0,0373435
ANKRD1	0,352501	0,0327467
PNP	0,352423	0,0401306
C12orf57	0,334051	0,0442766
PLS3	0,33323	0,0427922
RPL36AL	0,332242	0,045023
RSL24D1	0,326269	0,0409
SFRP1	-0,335853	0,0466334
ZBED4	-0,338071	0,045023
JAG1	-0,338471	0,0316437
MYBL2	-0,342803	0,0373435
RPRD2	-0,346317	0,0476626
IGSF3	-0,350148	0,038445
TGM2	-0,352487	0,0427922
FOXO3	-0,353689	0,0476626
AHNAK	-0,356798	0,0297104
FLNB	-0,357474	0,0409
CD81	-0,3575	0,0401306
RFX7	-0,358035	0,0419519
UHRF1	-0,361317	0,0316437
HPCAL1	-0,362478	0,0485774
LDOC1L	-0,363352	0,0239927

PIM3	-0,362381	0,0153371
TGFBRAP1	-0,363487	0,0350833
ZBTB9	-0,363858	0,0275868
CBX2	-0,364758	0,0429875
SLC35E2B	-0,364766	0,0327252
ASF1B	-0,365165	0,0320797
YWHAG	-0,365849	0,0105503
UBTD2	-0,366706	0,011309
STEAP3	-0,368777	0,0355872
TNFRSF1A	-0,369337	0,0492892
PAPSS2	-0,370356	0,0333646
TPBG	-0,370932	0,0168687
PATZ1	-0,372965	0,0288277
AKIRIN2	-0,37301	0,0153371
UBA1	-0,374506	0,0160813
ASXL2	-0,375562	0,011309
KLF9	-0,375662	0,0184004
HNRNPA0	-0,376856	0,00680068
PPARD	-0,377281	0,044652
IER3	-0,377481	0,00381732
SERINC2	-0,37767	0,0264419
PPP1R26	-0,378339	0,0288277
ZBTB40	-0,378806	0,0191454
INPP5E	-0,378888	0,0350833
MAPKAPK2	-0,379353	0,0128531
USP11	-0,379376	0,0236514
KANSL3	-0,380556	0,0392928
SGPL1	-0,381423	0,0487479
DHX30	-0,381487	0,0288277
VANGL2	-0,38184	0,0399584
SF3B4	-0,382331	0,0302768
SIK1	-0,382378	0,0207619
BICD2	-0,383466	0,011309
PPP1R15A	-0,385291	0,011309
TRAK1	-0,385569	0,0435682
ASB6	-0,385687	0,0339714
HIPK1	-0,386375	0,0120659
DNAJB12	-0,387423	0,0355872
TPRG1L	-0,387609	0,00381732
KHSRP	-0,388241	0,0418131
FSCN1	-0,388492	0,00965725
DRAXIN	-0,389236	0,0452196
RP5-935K16.1	-0,391738	0,0168687
SCUBE3	-0,392752	0,0236514
B4GALT1	-0,393778	0,00776792

ASXL2	-0,369463	0,0262606
NDOR1	-0,369748	0,036645
VAV2	-0,37176	0,0274911
INPP5E	-0,372213	0,0485774
CLIC6	-0,374103	0,0307295
MEPCE	-0,37506	0,0345094
ASF1B	-0,379056	0,0401306
DNER	-0,379948	0,0307295
SLC35E2B	-0,381628	0,0345094
FSCN1	-0,38391	0,0130136
DYNLL2	-0,384506	0,0142472
DNMT3B	-0,385622	0,0435674
KIAA1644	-0,385779	0,022904
MMP14	-0,386711	0,0373435
PRKACA	-0,387275	0,0435674
DUSP7	-0,387909	0,0239927
HSPA1B	-0,387968	0,0072481
CBX2	-0,388084	0,045023
SMO	-0,388665	0,0466334
ZNF318	-0,390694	0,0130136
ZHX2	-0,390877	0,0239927
SLC27A4	-0,396221	0,019501
ADAMTS5	-0,399077	0,0142472
SERINC5	-0,400801	0,0442766
ITGB3	-0,400967	0,0345094
TNFRSF1A	-0,402183	0,0427922
AC009299.4	-0,402717	0,0239927
CD276	-0,402959	0,0442766
VPS18	-0,403911	0,0116711
TBC1D13	-0,405612	0,00875918
MAP7D1	-0,405935	0,0442766
SLC45A3	-0,406356	0,0218788
FHDC1	-0,408776	0,036645
YTHDF1	-0,408915	0,0130136
SMAD3	-0,40984	0,0339678
RAB3B	-0,410985	0,0116711
SYMPK	-0,411177	0,0316437
TP53	-0,411181	0,0207525
EIF4EBP2	-0,411805	0,00213657
SMCR8	-0,412467	0,0102076
CHST14	-0,413971	0,0102076
CTC-503J8.6	-0,415809	0,0130136
SASH1	-0,41658	0,0116711
LOXL2	-0,416768	0,0401306
DNMBP	-0,417453	0,0130136

DUSP1	-0,395148	0,00776792
ZNF746	-0,395351	0,0320797
RELB	-0,395673	0,0412193
SNAPC4	-0,39586	0,0048506
SMAD7	-0,397832	0,0282818
PHLDA2	-0,399231	0,0191454
NKRF	-0,399943	0,0160813
NOL6	-0,400053	0,0048506
CYB5R3	-0,400122	0,00680068
SERPINA1	-0,401312	0,00381732
EIF1	-0,401364	0,0315796
PDE10A	-0,402777	0,0288277
MYO1C	-0,402842	0,0048506
CA12	-0,40285	0,0435682
FAM117B	-0,40322	0,0288277
MUL1	-0,403933	0,00381732
WASF3	-0,403957	0,0120659
PTPRN2	-0,40448	0,00871429
FAM101B	-0,404767	0,0048506
AP1B1	-0,405018	0,00583817
LPCAT1	-0,405107	0,00680068
GDI1	-0,405498	0,0120659
TRAM2	-0,405753	0,0215294
HMGA1	-0,405795	0,00381732
TNS3	-0,40667	0,0249688
ULBP3	-0,406791	0,0229553
SNAI1	-0,406824	0,0344907
SOX11	-0,407327	0,00267331
WDR62	-0,407535	0,0399584
PLEKHM3	-0,408222	0,044177
MOGS	-0,408553	0,011309
SV2A	-0,408766	0,00148451
MURC	-0,408864	0,0327252
PAQR7	-0,409356	0,00680068
DHX37	-0,409902	0,0191454
LOXL2	-0,410283	0,0339714
AXIN1	-0,410492	0,0315796
GJA3	-0,41125	0,0405302
PPP1R9B	-0,412778	0,0320797
RAC2	-0,413358	0,00381732
WBP2	-0,415221	0,00381732
MAP3K11	-0,415292	0,0236514
SURF4	-0,415588	0,00267331
RN7SL2	-0,416177	0,0288277
HYOU1	-0,416326	0,00871429

HIP1	-0,418486	0,045023
GPR161	-0,418752	0,0262606
EN2	-0,419048	0,0297104
NUP188	-0,419073	0,0218788
NCS1	-0,421133	0,0056613
PAK4	-0,421332	0,0056613
ZNF335	-0,421745	0,0130136
RP1-178F10.3	-0,421936	0,0218788
MAP2K3	-0,422324	0,0167771
ABTB2	-0,422434	0,0072481
SHISA2	-0,422534	0,0393926
SEPT9	-0,422771	0,036645
BRPF3	-0,423104	0,0401306
SPATA2	-0,425707	0,0427922
TMEM185B	-0,427254	0,0102076
SIGLEC15	-0,427647	0,0373435
CNN2	-0,427873	0,0218788
PTPRG	-0,428326	0,0409
MLLT1	-0,429595	0,0072481
ARAP3	-0,429801	0,025121
SSH1	-0,431116	0,0345094
FAM127C	-0,432022	0,0307295
CDCP1	-0,43429	0,00213657
LIMK1	-0,435476	0,00213657
HS6ST1	-0,435776	0,0180821
KIRREL	-0,437122	0,00213657
GBF1	-0,438222	0,00213657
KDM2A	-0,438455	0,0207525
KIAA1462	-0,44001	0,00213657
FAM117B	-0,440368	0,0156083
SATB2	-0,440971	0,0142472
PANX2	-0,441105	0,0393926
NPTX2	-0,44192	0,0167771
THSD4	-0,442852	0,022904
NES	-0,443995	0,0180821
ITGA11	-0,444329	0,0297104
EPHB6	-0,446041	0,0167771
PTK7	-0,446297	0,0427922
PIM2	-0,448043	0,019501
PHRF1	-0,448685	0,0156083
UBE2R2	-0,448891	0,0142472
FOXG1	-0,450804	0,0056613
WASF2	-0,452708	0,00213657
WNT3	-0,453425	0,0373435
HSPA1A	-0,455072	0,00213657

CHST2	-0,41638	0,0105503
ZMYM3	-0,418507	0,0380385
FAM20C	-0,419414	0,0176579
PLA2G3	-0,419441	0,0249688
PROX1	-0,421977	0,0176579
FZD8	-0,422118	0,00680068
NFKB1	-0,42222	0,00267331
WFS1	-0,422474	0,0269749
RASSF1	-0,422732	0,0160813
DLL1	-0,422908	0,0295788
ZSWIM6	-0,423011	0,0048506
PHLDA3	-0,423633	0,0405302
ABL2	-0,424035	0,014489
KLC2	-0,42434	0,0120659
GRN	-0,424578	0,011309
WDR26	-0,424641	0,036194
CERK	-0,42619	0,0207619
IER2	-0,426307	0,0487479
LINC00205	-0,426612	0,0160813
CLDN11	-0,427544	0,0309692
EXD3	-0,427702	0,0380385
NCS1	-0,427716	0,00148451
IGSF3	-0,428078	0,00267331
CTD-2044J15.2	-0,429359	0,0229553
FOXC2	-0,429363	0,0191454
PHC2	-0,429726	0,0048506
SLC16A2	-0,430799	0,00381732
GFPT2	-0,431737	0,00267331
NOMO2	-0,432267	0,0264419
WASF2	-0,432417	0,00148451
IGSF8	-0,433804	0,00680068
CLPTM1	-0,436084	0,0344907
MLLT1	-0,436292	0,00583817
ALKBH5	-0,436596	0,00148451
AXL	-0,436781	0,00148451
PPM1H	-0,436911	0,0048506
DNAJC6	-0,436912	0,0221892
B4GALT5	-0,437116	0,00267331
YTHDF1	-0,437474	0,00148451
CHSY1	-0,437517	0,00381732
GAS2L1	-0,437922	0,00776792
RP11-284F21.9	-0,438219	0,0215294
FITM2	-0,43841	0,00680068
TRAF1	-0,439017	0,0160813
IP6K1	-0,439832	0,0128531

TMEM178B	-0,455434	0,0130136
TGFB1I1	-0,456363	0,0262606
ECM1	-0,457132	0,00397639
NUDT19	-0,457755	0,0072481
UPF1	-0,457791	0,00397639
CSF3	-0,459683	0,022904
RIPK4	-0,460519	0,00213657
RIMS4	-0,461565	0,0156083
RNF122	-0,462136	0,0297104
LARP1	-0,462843	0,0345094
BCAR1	-0,464459	0,0102076
HIC2	-0,465095	0,0116711
MIER2	-0,465219	0,00213657
FAM155B	-0,466215	0,0239927
URB1	-0,466407	0,00397639
TPCN1	-0,468901	0,0466334
FZD5	-0,470179	0,0316437
MYBBP1A	-0,470599	0,0401306
EFNB2	-0,471724	0,00397639
S1PR3	-0,472258	0,00213657
BAG3	-0,473239	0,00213657
EPHB2	-0,473784	0,00213657
TFAP2C	-0,473908	0,0056613
GEMIN4	-0,474268	0,0142472
SPRED2	-0,474852	0,0297104
AGPAT1	-0,475342	0,036645
ZNF865	-0,477703	0,00397639
MYH9	-0,47772	0,00213657
MAVS	-0,480385	0,0102076
SMTN	-0,481465	0,00397639
SOX9	-0,481862	0,00213657
ARHGAP35	-0,482515	0,0056613
QSER1	-0,48261	0,0102076
PCDHB8	-0,483657	0,0393926
RP11-297M9.2	-0,48387	0,0102076
TOB2	-0,48415	0,0056613
FLNA	-0,48425	0,0207525
SP6	-0,485656	0,0102076
CSK	-0,486743	0,00397639
C17orf96	-0,488057	0,00875918
ITPKB	-0,488226	0,0056613
KIAA1244	-0,488523	0,00213657
EPN1	-0,488773	0,00875918
OTUB2	-0,488845	0,0207525
RP11-253E3.3	-0,489694	0,00397639

RNF122	-0,440263	0,0282818
RP11-297M9.2	-0,440353	0,0136673
GAL	-0,440592	0,00267331
INF2	-0,440693	0,0215294
ANKS1A	-0,441431	0,00680068
CRIPAK	-0,442339	0,0257637
TAOK2	-0,442554	0,0048506
BCL7A	-0,443192	0,014489
TRIB1	-0,444244	0,00583817
WDR4	-0,444714	0,0048506
EPN1	-0,444922	0,011309
FLT1	-0,445627	0,0120659
CDR2L	-0,446262	0,00267331
TICRR	-0,446586	0,0315796
ACTN1	-0,447325	0,00871429
PQLC1	-0,447936	0,0355872
CCDC134	-0,448281	0,0215294
ELL	-0,449345	0,011309
AFAP1	-0,449513	0,011309
FOXO3	-0,449763	0,00148451
LOX	-0,449907	0,0295788
CALB2	-0,450543	0,0327252
CRKL	-0,451522	0,00148451
TP53	-0,452016	0,00965725
HK2	-0,45213	0,00148451
CLIP2	-0,452579	0,0412193
C3orf52	-0,453859	0,0221892
ZNF70	-0,453982	0,0136673
RP11-698N11.4	-0,454947	0,0153371
RNF19B	-0,454953	0,00267331
SLC2A1	-0,454998	0,0243368
TINAGL1	-0,455184	0,0405302
SOBP	-0,455543	0,0477563
ZBTB7A	-0,457745	0,0350833
VPS18	-0,45795	0,00148451
MIR24-2	-0,4584	0,0048506
MAP2K1	-0,45867	0,0221892
WDR37	-0,458961	0,0168687
TFE3	-0,459148	0,00965725
TSHZ1	-0,459539	0,00148451
CTSD	-0,460866	0,00148451
TRIM11	-0,461136	0,0168687
KIAA1217	-0,46135	0,0418131
SYMPK	-0,461569	0,00583817
OTUB2	-0,462277	0,0275868

ATP6AP1	-0,491061	0,00213657
VARS	-0,49197	0,0072481
MAP3K9	-0,493226	0,00213657
AMIGO1	-0,493553	0,0130136
PML	-0,493833	0,0345094
RBPMS	-0,494407	0,0373435
WDTC1	-0,495415	0,00213657
RARG	-0,495428	0,0239927
CCDC120	-0,496342	0,045023
AP000350.5	-0,496392	0,0167771
FAM83H	-0,496409	0,0116711
FAM171A1	-0,496489	0,00213657
TOR4A	-0,496608	0,0056613
PROX1	-0,496656	0,0142472
CAPN5	-0,497228	0,0485774
FAM168A	-0,499653	0,0218788
SLC16A2	-0,501057	0,00213657
NAT8L	-0,501387	0,00397639
KIAA1217	-0,503343	0,0307295
CDIP1	-0,508111	0,00397639
PURB	-0,508363	0,00213657
TAPBP	-0,508481	0,00213657
FLJ00418	-0,509565	0,0435674
GPR63	-0,510074	0,0316437
SLX4	-0,510293	0,0072481
COL5A3	-0,510833	0,00213657
RP11-744K17.9	-0,510895	0,00213657
PARP16	-0,511175	0,0466334
MOB3A	-0,511542	0,00213657
MARCH4	-0,51197	0,00213657
MEGF9	-0,512444	0,00213657
NFATC4	-0,51422	0,0442766
BRI3BP	-0,514223	0,00213657
ATP6V0C	-0,514558	0,0056613
CDC25B	-0,514667	0,00213657
TGFBR3	-0,515913	0,00397639
TICRR	-0,517121	0,0102076
PRKCSH	-0,517778	0,00875918
SIRPA	-0,518298	0,00213657
CTD-2231H16.1	-0,519584	0,0307295
PHF8	-0,520835	0,00875918
RAD23A	-0,521364	0,00213657
ASTN1	-0,522127	0,0180821
RP11-989E6.10	-0,522189	0,0056613
TET3	-0,522304	0,0072481

MYO10	-0,462642	0,0168687
ADAM23	-0,46285	0,0418131
MINK1	-0,463026	0,0418131
FOXD2-AS1	-0,463092	0,0120659
ZNF335	-0,463481	0,00148451
UBE4B	-0,463482	0,0269749
C1orf233	-0,464506	0,0487479
FAM168A	-0,465261	0,0320797
EN2	-0,465417	0,00680068
DIP2B	-0,466178	0,00381732
KCTD12	-0,467313	0,0048506
MAVS	-0,4677	0,00680068
TNFRSF21	-0,467825	0,00148451
TGFB1I1	-0,468442	0,011309
AGPAT1	-0,469461	0,0288277
USP7	-0,470004	0,0120659
MEPCE	-0,470392	0,00148451
AC009299.4	-0,471295	0,00381732
RAD23A	-0,472329	0,00148451
PITPNC1	-0,473109	0,0269749
PQLC2	-0,474677	0,00583817
SRXN1	-0,475359	0,00148451
SMAD3	-0,475437	0,00776792
ZNF324B	-0,475667	0,0320797
TMEM127	-0,476244	0,00267331
CRTC1	-0,47773	0,00965725
SLC7A2	-0,477754	0,011309
CDC25B	-0,479187	0,0048506
VAX1	-0,482112	0,044652
ZNF185	-0,482603	0,0168687
GPC4	-0,483184	0,0269749
DAB2IP	-0,483198	0,00148451
MAFF	-0,483541	0,00267331
CANT1	-0,483632	0,00583817
TMEM185B	-0,483992	0,00148451
ELFN2	-0,484843	0,0327252
AP1M1	-0,484893	0,00267331
LINC00984	-0,486138	0,0344907
SERTAD3	-0,486142	0,0136673
AC079922.3	-0,487419	0,0184004
RP11-479G22.8	-0,487564	0,0160813
USP42	-0,4887	0,0048506
DNMT3B	-0,489949	0,00148451
RARA	-0,490686	0,00148451
MESDC1	-0,490777	0,00267331

C17orf103	-0,524614	0,00213657
ZBTB4	-0,525489	0,00213657
TNS3	-0,525914	0,00213657
KCTD12	-0,52616	0,00213657
PLXNB2	-0,526304	0,0274911
ARID1B	-0,526419	0,00213657
ARHGAP1	-0,527093	0,00213657
FRAS1	-0,528818	0,025121
SMOC1	-0,529058	0,00397639
KLHL21	-0,53173	0,00213657
POLR1A	-0,53218	0,0056613
LFNG	-0,533325	0,00213657
CABLES2	-0,536778	0,0167771
UBQLN2	-0,53735	0,00213657
COL1A2	-0,537776	0,0262606
NHSL2	-0,538819	0,0167771
SEMA6B	-0,542665	0,0466334
MYH14	-0,542835	0,00213657
SMARCA4	-0,543468	0,025121
BCL2	-0,544405	0,022904
FBN2	-0,544867	0,00213657
TSHZ3	-0,546342	0,00397639
FZD7	-0,54674	0,00213657
COL3A1	-0,547262	0,00213657
C11orf95	-0,547366	0,0116711
FAM20C	-0,54813	0,00213657
VIPR1	-0,548717	0,022904
NDST1	-0,549515	0,0274911
ZNF629	-0,550217	0,00213657
AC005943.5	-0,550658	0,00213657
CEP170B	-0,552379	0,022904
ZNF609	-0,55264	0,00213657
EPHB4	-0,552646	0,025121
ZMYM3	-0,553097	0,00213657
ARHGDIA	-0,553114	0,00397639
PLK1	-0,555517	0,00213657
GAS1	-0,555733	0,00213657
RP11-252A24.7	-0,556103	0,00213657
PLBD2	-0,556401	0,0102076
TBC1D10B	-0,557936	0,00213657
C3orf70	-0,558029	0,00397639
BAHD1	-0,558965	0,0239927
MBOAT7	-0,56	0,0072481
COL6A3	-0,560145	0,00213657
IRX2	-0,561424	0,0116711

ARSI	-0,491696	0,0295788
E2F2	-0,492146	0,0418131
ABR	-0,492387	0,0160813
LINC00969	-0,493779	0,0423086
GPC1	-0,493954	0,0350833
TMCC3	-0,494048	0,0249688
PALD1	-0,495475	0,0423086
ZNRF3	-0,495528	0,00680068
MAFG	-0,495578	0,0168687
C17orf96	-0,495809	0,00148451
ITPKB	-0,496999	0,00148451
BRI3BP	-0,497545	0,00148451
ARHGAP1	-0,498177	0,00148451
TSC22D4	-0,498246	0,00267331
PRKACA	-0,498437	0,00148451
ARHGAP35	-0,498614	0,0048506
CLCN4	-0,498662	0,0105503
COASY	-0,49882	0,0315796
LRRC4B	-0,499053	0,0153371
IMPDH1	-0,499161	0,00583817
ZNF341	-0,499979	0,00965725
SYNE3	-0,500536	0,00680068
CD276	-0,501509	0,00583817
PGF	-0,501809	0,0048506
WIPF2	-0,502154	0,0128531
ZNF275	-0,502939	0,00148451
COL4A1	-0,503026	0,0368232
EPHB2	-0,503246	0,00148451
FAM222A	-0,503298	0,0168687
ABTB1	-0,503713	0,0257637
COL6A3	-0,503807	0,00148451
SOX1	-0,504086	0,0105503
TYRO3	-0,504093	0,00267331
AMMECR1L	-0,504138	0,00148451
PARP16	-0,504786	0,0405302
ZNF503	-0,504843	0,0380385
ZNF629	-0,504997	0,00267331
KLHL29	-0,508476	0,00148451
DNAJC5	-0,508655	0,00148451
TSC22D1	-0,509295	0,00148451
DPYSL4	-0,5094	0,0452196
DPP9	-0,509486	0,0249688
ULK1	-0,509569	0,0136673
SLIT3	-0,509585	0,0344907
FMN1	-0,509667	0,0380385

PPP6R1	-0,563174	0,00213657
VDR	-0,563241	0,00397639
USP22	-0,563958	0,0167771
TRIM28	-0,564348	0,0156083
COL6A1	-0,566265	0,00213657
MARVELD1	-0,568169	0,00213657
ZNF503	-0,568799	0,0102076
FN1	-0,569794	0,0316437
TEAD3	-0,570166	0,0102076
RP11-359E3.4	-0,572169	0,0460184
SLC23A2	-0,573094	0,0442766
SAMD1	-0,573377	0,00213657
SERTAD2	-0,573749	0,00213657
SUFU	-0,573911	0,00213657
MYPOP	-0,574157	0,00213657
GPC4	-0,574995	0,00875918
ZFP36L1	-0,575275	0,00213657
RXRA	-0,576001	0,0072481
KANK2	-0,576933	0,0102076
PIAS3	-0,578734	0,00213657
BRD3	-0,579298	0,0116711
LRRC4B	-0,580077	0,0056613
WWC3	-0,580604	0,00875918
PLXNA1	-0,581955	0,00213657
SAMD4B	-0,582458	0,019501
TULP4	-0,582857	0,00213657
ERVWE2	-0,583116	0,00213657
LRP10	-0,584146	0,0130136
RP11-67L2.2	-0,585233	0,0142472
TRIM62	-0,585287	0,00213657
SPRED1	-0,585352	0,0167771
RP11-1246C19.1	-0,585358	0,00213657
STK35	-0,586551	0,00213657
TSHZ1	-0,587234	0,00213657
DLG5	-0,588571	0,00213657
FGFRL1	-0,58868	0,00213657
DMBX1	-0,589162	0,0327467
ZNF592	-0,589755	0,00213657
ANPEP	-0,5899	0,0156083
MARK2	-0,59081	0,00213657
LZTS1	-0,590881	0,00213657
CLUH	-0,590979	0,0262606
MAFB	-0,592972	0,0072481
LINC00565	-0,593991	0,00213657
CREB3L1	-0,595292	0,0180821

GPRIN1	-0,511955	0,00267331
ZC3H12C	-0,5134	0,00148451
MYBBP1A	-0,513498	0,0184004
GADD45G	-0,513533	0,00583817
KCTD5	-0,514584	0,00148451
SLC7A8	-0,514648	0,036194
TTC7B	-0,515033	0,0483062
SEPT9	-0,515096	0,00267331
ELK1	-0,515368	0,00148451
SGK223	-0,51569	0,0105503
PPM1E	-0,516069	0,00267331
ZNF865	-0,516204	0,00148451
TRIM56	-0,51653	0,0339714
DOCK4	-0,516785	0,0487479
BAIAP2-AS1	-0,517172	0,00776792
SAMD1	-0,517681	0,00148451
NUDT19	-0,518311	0,00148451
MGLL	-0,518746	0,00680068
GEMIN4	-0,521	0,0048506
CSK	-0,521339	0,00148451
PHRF1	-0,521445	0,014489
OGDH	-0,522323	0,00148451
EHD3	-0,522827	0,00148451
MAP7D1	-0,523677	0,00267331
PLEKHO2	-0,524019	0,00148451
NUAK2	-0,524238	0,00148451
SLC27A4	-0,524586	0,00148451
SREBF2	-0,524627	0,00148451
KIRREL	-0,524638	0,00148451
SLX4	-0,524674	0,00583817
GRB10	-0,525123	0,0176579
ZBED4	-0,525287	0,00148451
CAPN5	-0,525825	0,044177
KIFC3	-0,52655	0,049862
PHLDA1	-0,52712	0,00148451
SYDE1	-0,527406	0,0221892
SOX9	-0,527489	0,00148451
IGDCC4	-0,527685	0,0374192
RFTN1	-0,527812	0,0136673
GCNT4	-0,528495	0,00148451
RP11-395P17.3	-0,528801	0,00148451
RP11-611L7.1	-0,528857	0,0048506
WWC1	-0,529789	0,0368232
ZC3H7B	-0,530011	0,00148451
CLDN1	-0,530811	0,0221892

SCUBE3	-0,595604	0,00213657
TMEM184B	-0,596801	0,00213657
AGO1	-0,598185	0,00213657
APCDD1L	-0,598911	0,00213657
PXDN	-0,60025	0,00397639
EXTL3	-0,60127	0,0130136
CRTC1	-0,601511	0,00213657
KIAA1671	-0,605591	0,00213657
FURIN	-0,605734	0,00213657
STIM1	-0,606501	0,0435674
PCDHB13	-0,606581	0,00213657
WIZ	-0,607503	0,00213657
RP11-572C15.6	-0,607925	0,0056613
ADAM19	-0,609323	0,00213657
PAPD7	-0,610066	0,00213657
AP5B1	-0,611303	0,00213657
SCAMP4	-0,611978	0,0355607
CCNF	-0,612409	0,00213657
OBSL1	-0,612604	0,0355607
BCL11B	-0,613472	0,00213657
COL4A2	-0,616057	0,00875918
HOXC12	-0,616201	0,00213657
LMTK2	-0,616413	0,00213657
SAP130	-0,616586	0,00213657
SH3PXD2A	-0,617232	0,00213657
CNTNAP1	-0,61728	0,0427922
WNT7B	-0,619322	0,00213657
ZNF488	-0,619559	0,022904
WNT9A	-0,622653	0,022904
KCNC4	-0,623128	0,00213657
ZNF618	-0,623266	0,00213657
LMX1B	-0,623829	0,0401306
RP11-709B3.2	-0,626752	0,0167771
H6PD	-0,628352	0,00213657
PTPRF	-0,628487	0,00213657
SH3BP1	-0,630755	0,00213657
KMT2B	-0,632809	0,00213657
CLSTN1	-0,632993	0,00213657
GPRIN1	-0,63946	0,00213657
ATXN2	-0,639857	0,0056613
TNC	-0,641282	0,00213657
VSTM4	-0,647277	0,00213657
GABRQ	-0,651786	0,00213657
TRERF1	-0,652904	0,00213657
ACVR2B	-0,653172	0,0345094

RARG	-0,531174	0,00776792
DPYSL2	-0,531315	0,00148451
CSRNP1	-0,53327	0,00267331
TSC22D2	-0,533531	0,0465434
UBQLN2	-0,534016	0,00148451
SLC16A13	-0,534187	0,0128531
SLC7A5	-0,534222	0,00148451
PRKCSH	-0,534752	0,00267331
ARAP3	-0,536497	0,00148451
ITGA11	-0,537275	0,00583817
CDKN1A	-0,537736	0,00148451
CCND3	-0,53776	0,00148451
IRS1	-0,538206	0,00583817
URB1	-0,538917	0,00148451
SH3BP1	-0,539785	0,0423086
MROH1	-0,539833	0,044177
GPR63	-0,540474	0,0136673
FAM155B	-0,540674	0,00381732
LPP	-0,540766	0,0128531
ATXN2	-0,54094	0,0207619
GPR176	-0,541145	0,00267331
SNPH	-0,541579	0,00267331
GPR115	-0,542448	0,00148451
ITGA2	-0,542647	0,00148451
A4GALT	-0,543117	0,0302768
GREM1	-0,543955	0,00148451
SATB2	-0,544527	0,00148451
RIMS4	-0,544542	0,00148451
N4BP3	-0,544978	0,00148451
CTC-297N7.8	-0,546042	0,00148451
ZHX2	-0,546647	0,00148451
CNNM4	-0,546931	0,00267331
ZXDC	-0,547112	0,00148451
TMCC2	-0,54761	0,00148451
SH3RF1	-0,547692	0,00381732
RELA	-0,547996	0,00148451
UNC5B	-0,548466	0,00148451
ATP8B3	-0,549092	0,0452196
CICP27	-0,549369	0,0120659
EXTL3	-0,549586	0,00871429
POLR1A	-0,549713	0,00267331
REEP4	-0,549834	0,00148451
ARRB1	-0,549919	0,0229553
ECM1	-0,549988	0,00148451
CAPN15	-0,550052	0,00148451

FHL3	-0,655386	0,00213657
XYLT1	-0,655448	0,00213657
SMAD6	-0,65629	0,022904
MICALL1	-0,657071	0,00213657
CBX6	-0,657541	0,00213657
IGF2R	-0,658073	0,00213657
CBL	-0,658258	0,00213657
FOXJ2	-0,660956	0,00213657
CASC7	-0,662166	0,00213657
ZC3H3	-0,665378	0,00213657
GATAD2B	-0,666472	0,00213657
THBS1	-0,666878	0,00213657
GAL3ST4	-0,667209	0,0409
IRS1	-0,667937	0,00213657
DAG1	-0,668629	0,00213657
NFATC1	-0,669568	0,00213657
ABCC1	-0,670284	0,00397639
BEND3	-0,671284	0,00213657
PCDHB16	-0,671445	0,00213657
AP000974.1	-0,674199	0,0130136
MMP15	-0,675722	0,00213657
EFS	-0,678581	0,00213657
ADRA2C	-0,680311	0,0130136
TNFRSF21	-0,681574	0,00213657
RSPO4	-0,681694	0,0180821
BCOR	-0,683973	0,00213657
GPR56	-0,68578	0,0072481
COL5A1	-0,685894	0,0156083
SNAI1	-0,686084	0,00213657
HOXA13	-0,687027	0,0316437
SPDYE6	-0,687215	0,00213657
TCF7L2	-0,688912	0,038445
E2F2	-0,68936	0,00213657
DGAT2	-0,689728	0,00213657
RP11-244O19.1	-0,689917	0,00213657
FOXD2-AS1	-0,690673	0,00213657
SP2	-0,69323	0,00213657
POMK	-0,694241	0,00213657
LRP5	-0,694251	0,00213657
CHST6	-0,694972	0,0156083
KIAA1549L	-0,695234	0,00213657
ABCD1	-0,700165	0,0130136
ICOSLG	-0,701455	0,00213657
SLC44A2	-0,703832	0,00875918
MGRN1	-0,703927	0,00397639

SMARCA4	-0,550084	0,0207619
TFPI2	-0,550733	0,00148451
ABTB2	-0,550774	0,00148451
AMOTL2	-0,550929	0,00148451
AP5S1	-0,551967	0,00381732
SIRPA	-0,552139	0,00148451
PXN	-0,552316	0,0120659
BAG3	-0,552635	0,00148451
GPR161	-0,552914	0,00148451
EPHB6	-0,553293	0,00148451
ZBTB4	-0,553366	0,00148451
VARS	-0,554545	0,00148451
TSHZ3	-0,555272	0,00148451
GLTPD1	-0,555845	0,00148451
ARNT2	-0,555857	0,0048506
TNFAIP2	-0,555942	0,014489
NFKB2	-0,556347	0,00148451
NTNG2	-0,556742	0,00583817
AC010336.1	-0,556744	0,00148451
LFNG	-0,556897	0,00148451
ATP6V0C	-0,556953	0,00583817
DYNLL2	-0,557349	0,00148451
SPRED1	-0,557349	0,0320797
LIMK1	-0,558506	0,00148451
PDPN	-0,558545	0,00148451
PHF8	-0,558761	0,00267331
CDK6	-0,559294	0,00148451
RP11-890B15.3	-0,55942	0,0048506
SLC30A1	-0,559506	0,00148451
FZD1	-0,560035	0,00148451
PCDHB16	-0,560151	0,00148451
VAV2	-0,560394	0,00148451
SLC25A44	-0,560397	0,00148451
JAG1	-0,561722	0,00148451
MGAT5	-0,56304	0,0405302
MIEF1	-0,564302	0,00148451
POMK	-0,564435	0,00148451
RP11-744K17.9	-0,564525	0,00148451
ZBTB39	-0,564695	0,00148451
ERVWE2	-0,564791	0,00148451
GMEB2	-0,565006	0,00148451
ARID1B	-0,565213	0,00148451
TGM2	-0,565882	0,00148451
DNER	-0,566276	0,00148451
NRP2	-0,566317	0,00148451

DUSP5P1	-0,705205	0,00875918
RPTOR	-0,706216	0,00397639
TOX2	-0,706815	0,0102076
GLIS3	-0,707208	0,00213657
INCENP	-0,709777	0,00397639
IQSEC1	-0,711973	0,00213657
SIDT2	-0,712877	0,0167771
SALL2	-0,713806	0,0072481
FBXL18	-0,716465	0,00213657
EHD2	-0,717774	0,00213657
BCL3	-0,725041	0,00213657
DLX2	-0,728451	0,00213657
SCAF1	-0,73114	0,00213657
CELSR1	-0,731797	0,0393926
ADORA1	-0,732659	0,00397639
FAM46B	-0,734652	0,0409
TRRAP	-0,73811	0,00213657
ZNF423	-0,739452	0,00397639
LRRC15	-0,74329	0,00213657
SETD1A	-0,746058	0,00213657
ATG2A	-0,749213	0,028669
PCDHB14	-0,752466	0,00213657
CXorf67	-0,7551	0,00213657
FOXK1	-0,755219	0,022904
PLAGL2	-0,756377	0,00213657
GPR153	-0,757367	0,00213657
DRAXIN	-0,758055	0,00213657
EFR3B	-0,761548	0,00213657
GLIS1	-0,769065	0,00213657
NACC2	-0,76931	0,00213657
C9orf172	-0,769928	0,0218788
TTYH3	-0,771239	0,00213657
LRRC55	-0,772868	0,00213657
SHROOM2	-0,773168	0,00213657
FAM189B	-0,773212	0,00213657
PREX1	-0,775721	0,00213657
RARA	-0,777756	0,00213657
PRRC2B	-0,778603	0,00213657
MRC2	-0,778744	0,00213657
ZCCHC14	-0,779572	0,00213657
MAMLD1	-0,780295	0,00213657
NUP210	-0,78115	0,00213657
TIMP2	-0,782913	0,00213657
TNS1	-0,785519	0,0409
DKFZP761J1410	-0,786757	0,00213657

KDM2A	-0,566714	0,00381732
TBC1D13	-0,566863	0,00148451
BRD3	-0,566973	0,00267331
PAK4	-0,567691	0,00148451
FLYWCH1	-0,568169	0,0264419
TGFBR3	-0,569259	0,00267331
CPEB4	-0,569486	0,0350833
PAPD7	-0,569581	0,00871429
OAF	-0,56969	0,00148451
MED15	-0,569714	0,00148451
CTC-503J8.6	-0,570033	0,00148451
TMEM158	-0,570225	0,0199666
WIZ	-0,57027	0,00148451
C17orf103	-0,570328	0,00148451
LUM	-0,570563	0,00148451
RSPO4	-0,570847	0,0339714
SLC39A13	-0,570863	0,00583817
ATP6AP1	-0,57126	0,00148451
CTD-2207P18.2	-0,571477	0,0249688
AC004840.9	-0,571597	0,0368232
SOCS7	-0,571914	0,0269749
DLG5	-0,573037	0,00148451
VPS37C	-0,573455	0,00148451
HIC2	-0,573566	0,00148451
PPP1R18	-0,574174	0,00267331
SCARB1	-0,574964	0,00776792
TNIP1	-0,575579	0,00267331
SBNO2	-0,576574	0,011309
SLC46A3	-0,576669	0,00148451
RASA3	-0,576839	0,00148451
TMEM156	-0,577107	0,00148451
AMIGO1	-0,577419	0,00148451
SLC9A3R2	-0,5783	0,00148451
TEAD3	-0,57869	0,0120659
VEGFC	-0,578738	0,00148451
NCLN	-0,578801	0,00148451
KDM5C	-0,579371	0,00267331
ACO1	-0,579444	0,00148451
CTC-205M6.5	-0,58016	0,0136673
BAG6	-0,58023	0,00776792
IGSF9	-0,580552	0,00267331
SCAP	-0,581041	0,011309
EPAS1	-0,582004	0,0184004
FHDC1	-0,582831	0,00148451
EPHB4	-0,582929	0,0136673

IRS2	-0,788145	0,00213657
CAD	-0,791458	0,0116711
RP11-381O7.6	-0,792535	0,0401306
RP11-1055B8.7	-0,7935	0,0239927
PRRC2A	-0,798871	0,00213657
PITPNM2	-0,800763	0,00213657
RASD2	-0,805611	0,00213657
RP11-548H3.1	-0,805682	0,028669
ALX4	-0,811769	0,00213657
ATP8B3	-0,812531	0,00213657
KIAA1549	-0,814213	0,00213657
IGSF9	-0,814769	0,00213657
TBKBP1	-0,814942	0,00397639
NLGN2	-0,815403	0,00213657
ZYX	-0,815894	0,00213657
PTPRS	-0,818619	0,00213657
SLIT3	-0,823368	0,00213657
KIAA1522	-0,823665	0,00213657
SEC16A	-0,825496	0,00213657
PACS1	-0,825732	0,00213657
NEURL1B	-0,826957	0,00213657
NID1	-0,828825	0,00213657
C15orf39	-0,830915	0,00213657
AC092171.4	-0,835273	0,00213657
SFRP2	-0,84043	0,00213657
NOTCH2	-0,843576	0,00213657
SGK223	-0,843848	0,00213657
MAP1S	-0,846052	0,00213657
RUNX3	-0,850138	0,00213657
ENG	-0,851782	0,00213657
CORO2B	-0,854391	0,00213657
ANKRD52	-0,857366	0,00213657
ARID1A	-0,857563	0,00213657
MLXIP	-0,858125	0,00397639
TENM2	-0,858259	0,00213657
SKI	-0,859323	0,00213657
MSI1	-0,860873	0,0327467
PIP5K1C	-0,863451	0,00213657
PLCD4	-0,865901	0,0116711
TNRC18	-0,868399	0,0180821
FLG	-0,87074	0,00213657
HIPK2	-0,872707	0,00213657
BRD4	-0,87625	0,00213657
PLEC	-0,877531	0,0142472
ZNF703	-0,88088	0,00213657

DAP	-0,583914	0,00148451
SEMA4D	-0,584116	0,00148451
KCNK5	-0,584244	0,00148451
LARP1	-0,584279	0,00148451
LRRC8A	-0,584494	0,00148451
GNA12	-0,585657	0,00148451
SGPP2	-0,585952	0,00148451
DYNC1H1	-0,586037	0,0048506
CYP1B1	-0,5869	0,00148451
FAM109A	-0,587113	0,00267331
ARHGDIA	-0,587754	0,00148451
SLC39A14	-0,587876	0,00148451
FAM214B	-0,588173	0,00148451
FZD5	-0,588605	0,00148451
CLIC6	-0,589001	0,00148451
GRINA	-0,589751	0,00871429
SLC18A2	-0,589765	0,00148451
H6PD	-0,590648	0,00148451
SPRED2	-0,591139	0,0160813
MS4A4E	-0,592332	0,00267331
DDI2	-0,592381	0,00148451
RXRA	-0,592614	0,00583817
QSOX1	-0,592778	0,00148451
ZNF574	-0,592933	0,00148451
UPF1	-0,593017	0,00148451
SEMA4C	-0,593044	0,00148451
SEMA7A	-0,593692	0,00267331
ARHGAP39	-0,594007	0,0243368
RP11-863P13.3	-0,595024	0,00267331
TNFSF9	-0,595992	0,00148451
GABRQ	-0,598166	0,00148451
ITPRIPL2	-0,598274	0,00776792
RP11-572C15.6	-0,598314	0,00267331
CCNK	-0,598846	0,0368232
GPR153	-0,59885	0,00148451
IRAK2	-0,599945	0,00148451
KIF26A	-0,600188	0,00583817
HEG1	-0,601589	0,0191454
PFKFB4	-0,602348	0,0249688
RP11-395B7.7	-0,603273	0,00148451
CHST6	-0,605602	0,0249688
TRIM8	-0,605643	0,00148451
WDTC1	-0,605712	0,00148451
ZC3H3	-0,606413	0,00148451
FAM129B	-0,606935	0,00148451

GLI2	-0,88112	0,0262606
TAF4	-0,883022	0,00397639
AC006547.14	-0,895671	0,0427922
CHST1	-0,900359	0,00213657
EP300	-0,902307	0,00213657
TLE3	-0,908921	0,00213657
SEZ6L2	-0,91399	0,00213657
ZFHX3	-0,922174	0,00213657
ARHGAP23	-0,924554	0,028669
IGFBP5	-0,932385	0,00213657
KCNJ2	-0,933032	0,0207525
ZNF469	-0,93466	0,00213657
SLC6A8	-0,938317	0,0207525
GLTSCR1	-0,940184	0,0307295
PTPN23	-0,943027	0,00213657
OLFML2A	-0,945983	0,00213657
TMEM86A	-0,946796	0,0116711
SYNPO	-0,949499	0,00213657
NPTXR	-0,951228	0,00213657
CREBBP	-0,952927	0,00213657
DISP2	-0,956198	0,0142472
CIC	-0,95679	0,00213657
RP1-74M1.3	-0,960979	0,00213657
ZMIZ1	-0,978536	0,00213657
RREB1	-0,979846	0,00213657
FLNC	-0,98025	0,045023
AL050303.1	-0,983032	0,0072481
FOXRED2	-0,983437	0,00213657
RNA5-8SP6	-0,984896	0,0373435
TP53INP2	-0,995004	0,0297104
TMEM201	-1,0052	0,00213657
CMIP	-1,0201	0,00213657
ATN1	-1,02136	0,00213657
MN1	-1,026	0,0435674
AHDC1	-1,02828	0,00213657
PCDHGC3	-1,03052	0,00213657
RAVER1	-1,03811	0,00213657
NFIX	-1,04693	0,00213657
PVRL1	-1,04783	0,00213657
SBK1	-1,06077	0,00213657
EP400	-1,082	0,00213657
POM121	-1,08232	0,00213657
NAV1	-1,11232	0,00397639
CSPG4	-1,11328	0,00213657
LRFN3	-1,12308	0,00213657

RASD2	-0,607289	0,00148451
SOX13	-0,607356	0,00776792
IRF2BPL	-0,607671	0,00148451
SLC35F6	-0,608291	0,0344907
CCNJL	-0,609056	0,00148451
COL1A2	-0,609528	0,0048506
COL3A1	-0,610623	0,00148451
CACNG8	-0,611575	0,00148451
NCKAP5L	-0,611897	0,00148451
SSH1	-0,612198	0,00148451
RNF44	-0,61229	0,0221892
C3orf70	-0,613537	0,00148451
HK1	-0,613662	0,00148451
SMOX	-0,614218	0,0487479
NCOA6	-0,615877	0,00583817
MEGF9	-0,617118	0,00148451
GBF1	-0,618343	0,00148451
TRERF1	-0,618569	0,00148451
PPP6R1	-0,619133	0,00148451
CACNA2D4	-0,619509	0,0423086
EHD1	-0,619741	0,00148451
LRP4	-0,62074	0,0269749
THBS1	-0,621187	0,0048506
ATP2A2	-0,622191	0,00148451
MAP3K9	-0,622245	0,00148451
PTPRG	-0,622973	0,00148451
CDH4	-0,624471	0,0160813
EXT1	-0,624856	0,00267331
LDOC1L	-0,624893	0,00148451
NFATC1	-0,625514	0,0105503
TMEM81	-0,627014	0,00148451
AGO1	-0,627538	0,00148451
RP11-95M15.2	-0,62756	0,00148451
MARVELD1	-0,627711	0,00148451
ABCA3	-0,627854	0,00776792
PPP1R10	-0,628273	0,0309692
TMEM159	-0,62946	0,00148451
CDC42EP2	-0,63059	0,00148451
FAM43A	-0,630957	0,00148451
SASH1	-0,630959	0,00148451
IGF2BP1	-0,631031	0,0048506
ABCD1	-0,632106	0,0176579
PCSK6	-0,632126	0,0249688
RP11-426C22.4	-0,63308	0,00267331
IL15RA	-0,633315	0,00148451

WNK2	-1,1378	0,00213657
MEGF8	-1,14078	0,00213657
FASN	-1,14408	0,00213657
FOXP4	-1,14786	0,00213657
MAP1A	-1,15099	0,00213657
NCOR2	-1,16068	0,00213657
BGN	-1,18291	0,0355607
SHANK3	-1,20527	0,00213657
NFIC	-1,20772	0,00213657
SETD1B	-1,22022	0,00213657
ANO7	-1,22271	0,038445
HCFC1	-1,22338	0,00213657
SRCAP	-1,22586	0,028669
NOTCH1	-1,25535	0,00213657
MAGEL2	-1,25832	0,00213657
CTC-786C10.1	-1,26404	0,0102076
SOGA1	-1,27127	0,00213657
RERE	-1,31177	0,00213657
PRR12	-1,32083	0,00213657
HSPG2	-1,33007	0,00213657
RN7SL3	-1,33391	0,00213657
IGF2	-1,39994	0,00213657
FAM71D	-1,44006	0,0435674
POLR2A	-1,45371	0,00213657
BCORL1	-1,47541	0,00213657
BCL9L	-1,49347	0,00213657
COL1A1	-1,56212	0,00213657
NOTCH3	-1,69768	0,00213657
RN7SL2	-2,07018	0,00213657
CTD-2328D6.1	-3,37794	0,00213657

FLNB	-0,633587	0,00148451
KCTD16	-0,63363	0,0269749
ZNF592	-0,633929	0,00148451
FLJ00418	-0,63525	0,00381732
CNOT3	-0,636401	0,0309692
MORC2	-0,638989	0,00148451
DAG1	-0,639408	0,00148451
RRBP1	-0,639967	0,00148451
SHISA2	-0,6403	0,00148451
MARCH3	-0,641255	0,00583817
CDIP1	-0,641276	0,00148451
TPCN1	-0,641488	0,00267331
DRP2	-0,642267	0,00148451
C19orf55	-0,642954	0,00871429
MIR146A	-0,642963	0,00148451
BEND3	-0,643118	0,00148451
AC096772.6	-0,643365	0,0288277
PXDN	-0,643441	0,00871429
CDC42EP1	-0,643918	0,00148451
TMEM119	-0,644159	0,00583817
LATS2	-0,645748	0,00148451
PDGFA	-0,645951	0,00148451
RP11-244O19.1	-0,64743	0,00148451
FHL3	-0,648004	0,00148451
PPIF	-0,648545	0,00148451
DUSP5	-0,648925	0,00148451
TBC1D16	-0,649256	0,0374192
DNMBP	-0,649633	0,00148451
RGMB	-0,649884	0,00148451
SPATA2	-0,650246	0,00148451
PITPNM2	-0,650317	0,00381732
PTPRS	-0,650714	0,00148451
MEFV	-0,651056	0,00267331
USP22	-0,651234	0,00148451
CPEB3	-0,651294	0,00871429
BCR	-0,651627	0,0264419
MYH14	-0,651809	0,00148451
EEF2K	-0,652508	0,00148451
ECE1	-0,652893	0,00148451
PPARGC1B	-0,653226	0,0184004
CLCN7	-0,653344	0,00381732
FZD7	-0,653461	0,00148451
HBEGF	-0,653774	0,00148451
RPAP1	-0,654611	0,00148451
CLUH	-0,656169	0,00965725

RP11-57H14.4	-0,656393	0,00148451
SMCR8	-0,656483	0,00148451
FAM219A	-0,657831	0,00148451
ITGB3	-0,658133	0,00148451
SERTAD2	-0,658723	0,00148451
TRIM62	-0,658854	0,00148451
SRM	-0,659218	0,00148451
PFKFB3	-0,659329	0,00148451
RP1-178F10.3	-0,659615	0,00148451
MYH9	-0,659764	0,00148451
SLC36A1	-0,659895	0,00680068
FOXG1	-0,66019	0,00148451
TAPBP	-0,661557	0,00148451
CDK5R1	-0,663045	0,0333646
C15orf52	-0,663674	0,0282818
MGAT5B	-0,664073	0,00267331
RP11-252A24.7	-0,664277	0,00148451
OBSL1	-0,664783	0,0048506
SALL2	-0,665135	0,011309
EFR3B	-0,666359	0,00148451
DACT1	-0,666852	0,00148451
PCSK9	-0,668063	0,0333646
CABLES2	-0,66812	0,00148451
COL5A1	-0,66824	0,0236514
CBX6	-0,668303	0,00148451
MON1B	-0,668556	0,00148451
MAML1	-0,669967	0,00148451
KIF1C	-0,671497	0,0128531
SLC25A22	-0,672077	0,0339714
ANTXR2	-0,672104	0,00148451
DUSP7	-0,672933	0,00148451
SLC2A6	-0,673362	0,00148451
FOSL1	-0,674115	0,00148451
THBS2	-0,674312	0,00148451
HS3ST3B1	-0,674503	0,00148451
COL6A1	-0,674913	0,00148451
ARMC5	-0,675089	0,00381732
CYP4F11	-0,675759	0,00267331
PCDHB6	-0,675908	0,00148451
CHST1	-0,676016	0,00148451
FBXL17	-0,676486	0,00148451
CHST3	-0,677011	0,00148451
FAM83G	-0,677414	0,00148451
LMX1B	-0,677626	0,0105503
TACC2	-0,678213	0,00381732

PLXNB2	-0,678678	0,00148451
ZNF618	-0,678723	0,00148451
ITGA5	-0,679314	0,00148451
MAP1B	-0,679924	0,00148451
WNT9A	-0,680823	0,00148451
PLK1	-0,681532	0,00148451
NUP210	-0,681551	0,00148451
LTBP2	-0,682728	0,0374192
MRC2	-0,683306	0,011309
MMP14	-0,684327	0,00148451
RP11-284F21.10	-0,684834	0,00148451
DUSP4	-0,685609	0,00148451
FOXQ1	-0,685738	0,00583817
ITPKA	-0,685971	0,014489
SUFU	-0,687225	0,00148451
ADAM19	-0,687453	0,00148451
TICAM1	-0,687622	0,00148451
ABCC1	-0,688497	0,00381732
PLAUR	-0,688524	0,00148451
ASAP1	-0,688782	0,00267331
CNTNAP1	-0,689259	0,0128531
FBN2	-0,689269	0,00148451
NXPH4	-0,689464	0,00267331
PURB	-0,690952	0,00148451
BCAR1	-0,691039	0,00148451
GGT8P	-0,691346	0,0120659
CTD-2194D22.4	-0,691535	0,0184004
FGFRL1	-0,691629	0,00148451
PER2	-0,691724	0,0269749
ELF4	-0,692703	0,00148451
ESAM	-0,694402	0,00267331
C9orf172	-0,695236	0,0302768
SLC45A3	-0,696891	0,00148451
DCN	-0,699459	0,0048506
ITPRIP	-0,699921	0,00148451
TULP4	-0,699953	0,00148451
SCG5	-0,700542	0,00148451
GATA3	-0,70258	0,00267331
SAMD4B	-0,70336	0,00267331
GAPDHP32	-0,703844	0,0221892
UBE2O	-0,704326	0,0207619
SOCS3	-0,704806	0,00148451
VDR	-0,7051	0,00148451
RP11-709B3.2	-0,705868	0,00267331
NTN1	-0,705984	0,00148451

CCNF	-0,705997	0,00148451
TOX2	-0,70628	0,00148451
DGCR2	-0,706707	0,00148451
MMP15	-0,70723	0,00148451
PACS1	-0,708008	0,00148451
HS6ST1	-0,70804	0,00148451
NLRP3	-0,708656	0,00148451
TBC1D10B	-0,71057	0,00148451
PLBD2	-0,711226	0,00148451
TLN1	-0,712759	0,0309692
PML	-0,712864	0,00381732
CLSTN1	-0,713273	0,00148451
MFSD2A	-0,714332	0,00148451
LIF	-0,714782	0,00148451
TRIM28	-0,714957	0,00267331
APCDD1L	-0,715906	0,00148451
KIAA1549	-0,716675	0,00148451
BCL11B	-0,716682	0,00148451
SLC37A2	-0,716765	0,0120659
RUSC2	-0,717571	0,00148451
EGFR	-0,718482	0,00148451
U82695.10	-0,718988	0,00267331
RP11-509E16.1	-0,719238	0,00148451
ZC3H12D	-0,72061	0,00965725
CELSR1	-0,721751	0,0355872
SAP130	-0,721923	0,00148451
FURIN	-0,722214	0,00148451
AOC2	-0,722986	0,00148451
KLHL26	-0,723194	0,00148451
C11orf95	-0,724378	0,00583817
MGRN1	-0,724522	0,00148451
TIE1	-0,724525	0,00148451
AP5B1	-0,725698	0,00148451
RAB11FIP5	-0,727486	0,00680068
BCL2	-0,729999	0,00148451
LAPTM5	-0,730253	0,00148451
LRFN1	-0,730705	0,00267331
ZNF609	-0,730713	0,00148451
RNPEPL1	-0,732469	0,0199666
RBPMS2	-0,734913	0,00148451
RP11-391M1.4	-0,736854	0,00148451
BRPF3	-0,738593	0,00148451
KIAA1671	-0,738864	0,00148451
VSTM4	-0,740261	0,00148451
TCF7L1	-0,740869	0,014489

FAM83H	-0,740924	0,00148451
LAT	-0,7411	0,0128531
MOB3A	-0,741873	0,00148451
CXorf67	-0,742978	0,00148451
CXCL2	-0,743041	0,00148451
FAM115C	-0,743284	0,0269749
MYPOP	-0,744353	0,00148451
NACC2	-0,745164	0,00148451
SRGAP1	-0,745966	0,011309
RUNX1	-0,746055	0,00148451
RPL24P2	-0,747051	0,00680068
PRPF8	-0,747116	0,0229553
SERPINE1	-0,748334	0,00148451
NAT8L	-0,749844	0,00148451
TTYH3	-0,750598	0,00148451
LRP10	-0,751808	0,00148451
MAMLD1	-0,752704	0,00148451
G0S2	-0,752756	0,00148451
FOXJ2	-0,753577	0,00148451
TRRAP	-0,754977	0,00148451
GLIS1	-0,756688	0,00148451
ATXN2L	-0,757216	0,00148451
PCDHB14	-0,758235	0,00267331
STK35	-0,758435	0,00148451
PODXL	-0,759089	0,00148451
ZC3H12A	-0,759943	0,00148451
LDLR	-0,761347	0,00148451
SCRIB	-0,762743	0,00871429
HPCAL1	-0,763827	0,00148451
SH2B3	-0,76387	0,00148451
RP11-21L23.2	-0,764722	0,00148451
EPHB1	-0,764789	0,00148451
TOB2	-0,764818	0,00148451
KIAA0247	-0,766302	0,00148451
CHPF2	-0,766459	0,00148451
SPHK1	-0,766919	0,00148451
C17orf51	-0,768819	0,00583817
C15orf48	-0,768823	0,00148451
KCNN3	-0,768982	0,00148451
NHSL2	-0,769818	0,00148451
LRRC15	-0,772273	0,00148451
RAB27B	-0,773421	0,00148451
SCAMP4	-0,77371	0,00148451
WNK1	-0,773975	0,00965725
HMOX1	-0,774257	0,00148451

COL5A3	-0,775788	0,00148451
VNN1	-0,775806	0,0199666
SH3RF3	-0,775849	0,00148451
PRRC2A	-0,776501	0,00267331
PLAU	-0,778695	0,00148451
PLXNA1	-0,781126	0,00148451
CASC7	-0,782321	0,00148451
SLC7A1	-0,782405	0,00148451
MARK2	-0,784961	0,00148451
ACVR2B	-0,786716	0,00680068
SPRY4	-0,787318	0,00148451
COL4A2	-0,787673	0,00148451
ONECUT2	-0,789259	0,00148451
SHROOM2	-0,790136	0,00148451
IL6	-0,790652	0,00148451
AP001062.7	-0,79098	0,0105503
RP11-245M24.1	-0,791652	0,0269749
MIER2	-0,791813	0,00148451
XYLT1	-0,794618	0,00148451
PTPRF	-0,796996	0,00148451
FBRS	-0,797077	0,00680068
RUNX3	-0,798064	0,0215294
PAPPA	-0,798138	0,00148451
DUSP8P5	-0,798326	0,044177
TSSK5P1	-0,798762	0,0221892
PRRC2B	-0,799192	0,00148451
CEP170B	-0,799276	0,00148451
INCENP	-0,799345	0,00148451
EPHA2	-0,79961	0,00148451
TIMP2	-0,800318	0,00148451
C1orf106	-0,800356	0,00148451
SETD1A	-0,802872	0,00148451
SBF1	-0,804363	0,00148451
SYTL3	-0,804693	0,00381732
UCN2	-0,805114	0,0191454
KYNU	-0,806024	0,00148451
RP11-178G16.4	-0,80649	0,0191454
KIF1A	-0,809918	0,0160813
GATAD2B	-0,810165	0,00148451
FLNA	-0,810283	0,00148451
BAHD1	-0,810752	0,00148451
DTX2	-0,811929	0,00148451
RP11-381O7.6	-0,81319	0,0191454
CDCP1	-0,813191	0,00148451
EGR1	-0,813453	0,00148451

RP11-211G23.2	-0,814018	0,0344907
SLCO4A1	-0,814191	0,00148451
WWC3	-0,816863	0,00148451
CTC-786C10.1	-0,817881	0,0392928
IGF2R	-0,818563	0,00148451
RP11-253E3.3	-0,821576	0,00148451
KCNJ12	-0,82301	0,00148451
CXCL3	-0,828139	0,00148451
SLC9A7	-0,83307	0,00381732
TNIP3	-0,834032	0,0128531
FAM189B	-0,837564	0,00148451
NID1	-0,837899	0,00148451
C1QTNF1	-0,838228	0,00148451
FOXK1	-0,839425	0,0048506
TAF4	-0,83951	0,0136673
ZNF703	-0,840682	0,00148451
KIAA1644	-0,842912	0,00148451
AC003092.1	-0,842963	0,00148451
LMTK2	-0,843003	0,00148451
SCAF1	-0,843245	0,00148451
BCOR	-0,845814	0,00148451
SMTN	-0,84685	0,00148451
PAPPA-AS1	-0,847822	0,0320797
LRFN3	-0,848068	0,0120659
MLXIP	-0,850742	0,00381732
SELPLG	-0,852412	0,0320797
MICALL1	-0,853116	0,00148451
DUSP5P1	-0,853444	0,00148451
SIGLEC15	-0,853721	0,00148451
CCND1	-0,853902	0,00148451
ADCY9	-0,853997	0,0048506
INA	-0,85705	0,00148451
DLX2	-0,858222	0,00148451
RPTOR	-0,859105	0,00148451
TMEM184B	-0,859425	0,00148451
FAM171A1	-0,86004	0,00148451
CYP1A1	-0,862029	0,0264419
NDST1	-0,862385	0,00148451
TNC	-0,865641	0,00148451
CAD	-0,873411	0,00148451
SLAMF7	-0,873808	0,0435682
SF3A1	-0,873989	0,0315796
IQSEC1	-0,87522	0,00148451
NR4A3	-0,877342	0,00148451
ENG	-0,882301	0,00148451

SH3PXD2B	-0,882329	0,00583817
LPHN1	-0,882671	0,0269749
ABCA1	-0,885593	0,00965725
THSD4	-0,885603	0,00148451
TET3	-0,885806	0,00148451
ARHGEF37	-0,886979	0,00381732
KMT2B	-0,887819	0,00148451
SIPA1L3	-0,888493	0,0176579
FMNL1	-0,888692	0,00267331
SLC6A8	-0,890029	0,0249688
AC112721.1	-0,890877	0,00148451
FAM65A	-0,892547	0,0269749
CBL	-0,893648	0,00148451
AC005943.5	-0,894424	0,00148451
SYNPO	-0,897113	0,00148451
IGFN1	-0,898636	0,00148451
RP11-134G8.8	-0,902642	0,0120659
MAFB	-0,902832	0,00148451
CREB3L2	-0,903555	0,00148451
NPTXR	-0,90497	0,00148451
KIAA1549L	-0,906117	0,00148451
RP5-1042I8.7	-0,906971	0,011309
CYGB	-0,907903	0,00148451
PIM2	-0,908957	0,00148451
SLC9A1	-0,911132	0,00267331
POU2F2	-0,912689	0,0120659
ZNF628	-0,913493	0,00381732
FOXF1	-0,9138	0,00148451
STC1	-0,915743	0,00148451
EHD2	-0,91586	0,00148451
SEPN1	-0,92024	0,0492892
LRIG1	-0,921489	0,00148451
PLAGL2	-0,923836	0,00148451
CCL20	-0,92504	0,00148451
PDGFRB	-0,926227	0,014489
MIDN	-0,926304	0,00148451
AC112721.2	-0,92654	0,00148451
RP11-1055B8.7	-0,92662	0,0105503
TOR4A	-0,929864	0,00148451
MEF2D	-0,930961	0,00148451
TFAP2C	-0,93373	0,00148451
AMPD3	-0,934328	0,0048506
ATN1	-0,936254	0,00148451
AC002480.3	-0,940706	0,0176579
AHNAK2	-0,94071	0,00267331

LINC00565	-0,941773	0,00148451
ITPR3	-0,943077	0,00148451
TENM2	-0,943439	0,00148451
RAB3B	-0,94448	0,00148451
C3	-0,946004	0,00148451
LINC00341	-0,956922	0,0221892
PANX2	-0,959601	0,00148451
TMEM132A	-0,960421	0,0229553
MAPK7	-0,960571	0,0105503
CREBBP	-0,961195	0,00381732
C2CD4A	-0,962681	0,00148451
ATG2A	-0,964077	0,00148451
ZSWIM4	-0,965242	0,00148451
TLE3	-0,966681	0,00148451
MAP2K3	-0,96682	0,00148451
RP11-215G15.5	-0,967181	0,00148451
RP11-427H3.3	-0,967362	0,00871429
KIAA1244	-0,967865	0,00148451
FBXL18	-0,968115	0,00148451
NOTCH2	-0,968767	0,00148451
LINC00622	-0,971535	0,00148451
PLXNA2	-0,972516	0,00148451
TNFSF14	-0,972812	0,00871429
ARHGAP23	-0,974967	0,0128531
FOXRED2	-0,977705	0,00148451
SP2	-0,979141	0,00148451
RP11-548H3.1	-0,980516	0,0048506
SOD2	-0,980661	0,011309
LUCAT1,RP11-213H15.4	-0,982252	0,0184004
KCND1	-0,986944	0,014489
ZYX	-0,990679	0,00148451
SH3PXD2A	-0,991329	0,00148451
PAX8-AS1	-0,993437	0,0048506
PLCD4	-0,994046	0,0199666
ZCCHC14	-0,994428	0,00148451
HELZ2	-0,998247	0,00776792
ZNF142	-0,99838	0,0387115
RP11-83N9.5	-0,998553	0,0128531
SBK1	-1,00235	0,00148451
GPR68	-1,00429	0,00148451
ANKRD52	-1,00442	0,00148451
BRD4	-1,01039	0,00680068
SLC47A1	-1,01184	0,036194
KLHL21	-1,01643	0,00148451
SPP1	-1,01794	0,00148451

MARCH4	-1,01919	0,00148451
RREB1	-1,02154	0,0160813
NKD1	-1,02932	0,00583817
HSD3BP5	-1,02955	0,00148451
IRS2	-1,02963	0,00148451
KCNC4	-1,03208	0,00148451
RP1-74M1.3	-1,03279	0,00148451
KIAA1522	-1,03398	0,00148451
AHDC1	-1,03441	0,00148451
MAP1S	-1,03777	0,00148451
GDNF	-1,04056	0,00148451
SKI	-1,04468	0,00148451
C4orf26	-1,04526	0,00381732
SEZ6L2	-1,04963	0,00148451
PIP5K1C	-1,05077	0,00148451
PIK3CD	-1,05423	0,00267331
SPATA13	-1,05537	0,0184004
LINC00520	-1,05652	0,00148451
WNK2	-1,05912	0,00148451
NFIX	-1,06191	0,00148451
PTPN23	-1,0639	0,00148451
ARID1A	-1,06639	0,00148451
EP300	-1,06685	0,00148451
NFIC	-1,0678	0,00148451
BCL3	-1,06946	0,00148451
TMEM201	-1,07029	0,00148451
AKR1C2	-1,07095	0,0483062
CELSR2	-1,07448	0,00148451
GLI2	-1,07782	0,00148451
CXCL1	-1,07812	0,00148451
SMOC1	-1,07943	0,00148451
PAX1	-1,07954	0,0048506
DKFZP761J1410	-1,08029	0,00148451
AL050303.1	-1,08267	0,00267331
SCARF1	-1,08506	0,00148451
PCDHGC3	-1,09397	0,00148451
TP53INP2	-1,10049	0,00381732
KIF21B	-1,10403	0,00148451
GLIS3	-1,10434	0,00148451
SEC16A	-1,10818	0,00148451
KLRC2	-1,11805	0,0243368
LAMB3	-1,11968	0,00148451
NR1D1	-1,12074	0,014489
C15orf39	-1,1237	0,00148451
C6orf58	-1,12411	0,00148451

COL13A1	-1,12488	0,00148451
PVRL1	-1,1321	0,00148451
RAVER1	-1,1336	0,00148451
SPEN	-1,13606	0,00148451
WNT7B	-1,14249	0,00148451
ZFHX3	-1,15392	0,00148451
SP6	-1,15625	0,00148451
RP11-1246C19.1	-1,16259	0,00148451
AC004257.3	-1,17063	0,0275868
DISP2	-1,17292	0,00148451
SH2D2A	-1,17717	0,00148451
C1orf226	-1,17846	0,0264419
C1orf204	-1,18084	0,0315796
AC068279.3	-1,18412	0,00267331
FLG	-1,19371	0,00148451
IL8	-1,19462	0,00148451
AC092171.4	-1,19525	0,00148451
TUBB4A	-1,19703	0,0105503
SLC6A12	-1,2006	0,0264419
CMIP	-1,2071	0,00148451
NAV1	-1,20879	0,00148451
EHF	-1,21193	0,014489
CORO2B	-1,2184	0,00148451
CSPG4	-1,22085	0,00148451
IL1A	-1,2219	0,00148451
PTGS2	-1,22207	0,00148451
RERE	-1,23754	0,00267331
ANPEP	-1,23876	0,00148451
CTD-2319I12.1	-1,24177	0,00148451
MMP9	-1,24345	0,00148451
MEGF8	-1,24461	0,00148451
HSPG2	-1,24637	0,00148451
SFRP2	-1,24871	0,00148451
CLDN14	-1,2511	0,00148451
ZMIZ1	-1,25251	0,00148451
KIAA1462	-1,25536	0,00148451
GAPDHP33	-1,25604	0,00148451
TNRC18	-1,26078	0,00148451
ADAM8	-1,26919	0,00148451
MMP1	-1,27298	0,00148451
RFX1	-1,27598	0,0243368
PLEC	-1,2765	0,00148451
EP400	-1,27864	0,00148451
NCOR2	-1,28453	0,00148451
NRXN2	-1,29082	0,0120659

MN1	-1,29428	0,00267331
CIC	-1,29724	0,00148451
FAM222B	-1,2986	0,0344907
FOXP4	-1,30235	0,00148451
SHANK3	-1,31369	0,00148451
SRCAP	-1,31375	0,00871429
LRRC55	-1,32429	0,00148451
ROBO4	-1,32924	0,00148451
FBRSL1	-1,33765	0,0355872
BCL2A1	-1,33889	0,00148451
HCFC1	-1,34007	0,00148451
NOTCH1	-1,34022	0,00148451
POM121	-1,34994	0,00148451
RP11-443P15.2	-1,35293	0,00148451
SETD1B	-1,35633	0,00148451
SPTBN4	-1,35981	0,0288277
FLNC	-1,36706	0,00148451
AKR1C1	-1,37128	0,00148451
BMPER	-1,38293	0,00148451
CLIP3	-1,388	0,0374192
ICOSLG	-1,39219	0,00148451
PREX1	-1,40264	0,00148451
HCK	-1,41135	0,00148451
HIPK2	-1,41879	0,00148451
CREB3L1	-1,42162	0,00148451
RP11-548O1.3	-1,43986	0,00871429
CXCL5	-1,45476	0,00148451
SOGA1	-1,47148	0,00148451
POLR2A	-1,48131	0,00148451
PRR12	-1,49271	0,00148451
GAPDHP27	-1,50731	0,0243368
LRP1	-1,52916	0,036194
CTD-2026K11.1	-1,53063	0,0483062
ASB2	-1,5572	0,00148451
IL1RN	-1,59525	0,00148451
BCORL1	-1,6034	0,00148451
IL11	-1,61109	0,00148451
GAREML	-1,62328	0,0221892
FASN	-1,62659	0,00148451
MAP1A	-1,63906	0,00148451
KDM6B	-1,64593	0,00148451
COL1A1	-1,64849	0,00148451
ZNF469	-1,73396	0,00148451
BCL9L	-1,75381	0,00148451
NOTCH3	-1,78432	0,00148451

AC144831.1	-1,78513	0,00148451
SERPINB2	-1,92272	0,00148451
FAM71D	-1,97223	0,0120659
RP11-907D1.2	-2,00065	0,00148451
MS4A7	-2,05459	0,011309
IL1B	-2,11157	0,00148451
SLCO2B1	-2,11709	0,00148451
AC069363.1	-2,17573	0,00148451
ESM1	-2,2405	0,00148451
IGF2	-2,25652	0,00148451
RP3-483K16.4	-2,26584	0,0465434
IL36RN	-2,42675	0,00148451
CSF3	-2,5118	0,00148451
CCL3	-3,09618	0,00148451
CTD-2328D6.1	-3,10765	0,00148451

Table S4: Verification of sample contamination using Kraken tool. K-mers were mapped to a pre-built 4 GB database constructed from complete bacterial, archaeal, and viral genomes in RefSeq. Special columns for *Mycoplasma* spp., *Flaviviridae* (TBEV) and *Betaherpesvirinae* (HCMV) included in bacteria and viruses section, respectively. Links for interactive results are located in column with replicate numbers.

sample	replicate	total reads	bacteria		viruses			archea	unnassigned
			total	*Mycoplasma* spp.	total	*Flaviviridae*	*Betaherpesvirinae*		
TBEV + IFN-β	#1	41734277	110825 (0,3%)	206	3789 (0,009%)	671	107	957 (0,002%)	7625 (0,02%)
	#2	40495494	82118 (0,2%)	407	3428 (0,008%)	529	60	863 (0,002%)	6875 (0,02%)
	#3	52489405	168355 (0,3%)	404	4865 (0,009%)	640	144	1218 (0,002%)	11163 (0,02%)
TBEV	#1	41637668	147588 (0,4%)	234	168860 (0,4%)	165302	88	1049 (0,003%)	7896 (0,02%)
	#2	47976602	123404 (0,3%)	193	202008 (0,4%)	198244	92	1039 (0,002%)	6163 (0,01%)
	#3	40129384	86895 (0,2%)	166	167206 (0,4%)	164453	53	767 (0,002%)	6339 (0,02%)
IFN-β	#1	48449811	68645 (0,1%)	231	4025 (0,008%)	148	143	12212 (0,03%)	8497 (0,02%)
	#2	94741973	85642 (0,09%)	442	7524 (0,008%)	195	230	3520 (0,004%)	14638 (0,02%)
	#3	39654999	305961 (0,8%)	210	3332 (0,008%)	101	70	5499 (0,01%)	7779 (0,02%)
mock	#1	41736063	185360 (0,4%)	240	3488 (0,008%)	119	102	2438 (0,006%)	7916 (0,02%)
	#2	39507000	308412 (0,8%)	238	3535 (0,009%)	94	99	14134 (0,04%)	8395 (0,02%)
	#3	47665479	120521 (0,3%)	212	4044 (0,008%)	121	99	1476 (0,003%)	8276 (0,02%)

Table S5: **Top 20 up/down-regulated genes detected in TBEV-infected DAOY cells compared to untreated mock cells.** For completeness, other comparative data lists are also included. The rank (if possible, otherwise N/A – not available) represents the position of the gene in the list of differentially expressed genes of respective dataset; fold-change for each gene in all three datasets is shown.

up-regulated genes		mock vs TBEV		mock vs IFN-β		mock vs TBEV+IFN-β	
gene	Entrez name	rank	fold change	rank	fold change	rank	fold change
IFNL1	interferon lambda 1	1	175.44	N/A	1	N/A	1
CXCL11	C-X-C motif chemokine ligand 11	2	30.11	N/A	1	N/A	1
CXCL10	C-X-C motif chemokine ligand 10	3	28.82	N/A	1	82	1.66
CCL4L2	C-C motif chemokine ligand 4 like 2	4	16.90	N/A	1	N/A	1
IFIT2	interferon induced protein with tetratricopeptide repeats 2	5	13.59	N/A	1	N/A	1
TAC3	tachykinin 3	6	11.47	N/A	1	N/A	1
SPRR2D	small proline-rich protein 2D	7	11.04	N/A	1	N/A	1
CCL5	C-C motif chemokine ligand 5	8	10.73	N/A	1	163	1.42
RANBP3L	RAN binding protein 3 like	9	9.06	N/A	1	N/A	1
IFIT1	interferon induced protein with tetratricopeptide repeats 1	10	9.01	N/A	1	N/A	1
RAET1L	retinoic acid early transcript 1L	11	8.51	N/A	1	N/A	1
RSAD2	radical S-adenosyl methionine domain containing 2	12	7.19	N/A	1	23	2.27
H19	H19, imprinted maternally expressed transcript (non-protein coding)	13	7.03	N/A	1	N/A	1
OASL	2'-5'-oligoadenylate synthetase like	14	6.92	N/A	1	200	1.38
KRT16	keratin 16	15	6.37	N/A	1	N/A	1
TAC1	tachykinin precursor 1	16	5.75	N/A	1	N/A	1
KRT14	keratin 14	17	5.64	N/A	1	40	2.00
SCN3A	sodium voltage-gated channel alpha subunit 3	18	5.27	N/A	1	N/A	1
RP11-757G1.6	long non-coding RNA	19	5.10	N/A	1	115	1.56
DDIT3	DNA damage inducible transcript 3	20	4.81	N/A	1	N/A	1

down-regulated genes		mock vs TBEV		mock vs IFN-β		mock vs TBEV+IFN-β	
gene	Entrez name	rank	fold change	rank	fold change	rank	fold change
CTD-2328D6.1	long non-coding RNA	1	0.10	65	0.59	1	0.12
RN7SL2	RNA, 7SL, cytoplasmic 2	2	0.24	40	0.48	1007	0.75
NOTCH3	NOTCH3	3	0.31	N/A	1	16	0.29
COL1A1	collagen type I alpha 1	4	0.34	N/A	1	19	0.32
BCL9L	B cell CLL/lymphoma 9-like	5	0.36	N/A	1	17	0.30
BCORL1	BCL6 corepressor-like 1	6	0.36	N/A	1	25	0.33
POLR2A	polymerase (RNA) II subunit A	7	0.37	N/A	1	32	0.36
FAM71D	family with sequence similarity 71 member D	8	0.37	N/A	1	13	0.25
IGF2	insulin like growth factor 2	9	0.38	N/A	1	6	0.21
RN7SL3	RNA, 7 SL, cytoplasmic 3	10	0.40	N/A	1	N/A	1
HSPG2	perlecan (heparan sulfate proteoglycan 2)	11	0.40	N/A	1	74	0.42
PRR12	proline rich 12	12	0.40	N/A	1	31	0.36
RERE	arginine-glutamic acid dipeptide repeats	13	0.40	N/A	1	79	0.42
SOGA1	suppressor of glucose, autophagy associated 1	14	0.41	N/A	1	33	0.36
CTC-786C10.1	long non-coding RNA	15	0.42	N/A	1	282	0.57
MAGEL2	MAGE family member L2	16	0.42	N/A	1	N/A	1
NOTCH1	NOTCH1	17	0.42	N/A	1	49	0.39
SRCAP	Snf2-related CREBBP activator protein	18	0.43	N/A	1	55	0.40
HCFC1	host cell factor C1	19	0.43	N/A	1	50	0.40
ANO7	anoctamin 7	20	0.43	N/A	1	N/A	1

Table S6: Top 20 up/down-regulated genes detected in IFN-β-treated DAOY cells compared to untreated mock cells. For completeness, other comparative data lists are also included. The rank (if possible, otherwise N/A – not available) represents the position of the gene in the list of differentially expressed genes of respective dataset; fold-change for each gene in all three datasets is shown.

up-regulated genes		mock vs IFN-β		mock vs TBEV		mock vs TBEV + IFN-β	
gene	Entrez name	rank	fold change	rank	fold change	rank	fold change
IFI27	interferon alpha inducible protein 27	1	32.53	75	2.48	1	44.67
IFI6	interferon alpha inducible protein 6	2	18.52	146	1.79	2	26.76
RNA5-8SP6	RNA, 5.8S ribosomal pseudogene 6	3	12.58	836	0.51	20	2.36
RMRP	RNA component of mitochondrial RNA processing endoribonuclease	4	10.82	N/A	1	5	3.77
RN7SK	RNA, 7SK small nuclear	5	9.63	N/A	1	12	2.89
IFITM1	interferon induced transmembrane protein 1	6	9.21	N/A	1	3	12.76
AC010970.2	processed pseudogene	7	7.02	N/A	1	N/A	1
BST2	bone marrow stromal cell antigen 2	8	3.80	174	1.68	4	5.09
EPSTI1	epithelial stromal interaction 1 (breast)	9	2.72	38	3.56	6	3.52
RP5-1021I20.2	long non-coding RNA	10	2.66	N/A	1	11	3.00
AC187652.1	long non-coding RNA	11	2.41	N/A	1	N/A	1
RUNX1T1	RUNX1 translocation partner 1	12	2.25	79	2.43	29	2.16
CRABP2	cellular retinoic acid binding protein 2	13	2.25	N/A	1	14	2.59
RP11-260A9.6	long non-coding RNA chromosome 17	14	2.21	139	1.87	24	2.23
IFITM3	interferon induced transmembrane protein 3	15	2.11	N/A	1	13	2.64
ST8SIA6	ST8 alpha-N-acetyl-neuraminide alpha-2,8-sialyltransferase 6	16	2.07	N/A	1	N/A	1
RGCC	regulator of cell cycle	17	2.07	N/A	1	18	2.46
PADI3	peptidyl arginine deiminase 3	18	2.05	N/A	1	N/A	1
SERPING1	serpin family G member 1	19	2.02	N/A	1	N/A	1
CT45A5	cancer/testis antigen family 45, member A5	20	1.99	159	1.74	58	1.81

down-regulated genes		mock vs IFN-β		mock vs TBEV		mock vs TBEV + IFN-β	
gene	Entrez name	rank	fold change	rank	fold change	rank	fold change
IL36RN	interleukin 36 receptor antagonist	1	0.12	N/A	1	4	0.19
CCL3	C-C motif chemokine ligand 3	2	0.16	794	2.30	2	0.12
CSF3	colony stimulating factor 3	3	0.19	430	0.73	3	0.18
IL1B	interleukin 1 beta	4	0.22	N/A	1	10	0.23
ITGAX	integrin subunit alpha X	5	0.27	N/A	1	N/A	1
SLAMF8	SLAM family member 8	6	0.27	N/A	1	N/A	1
HCK	HCK proto-oncogene, Src family tyrosine kinase	7	0.30	N/A	1	38	0.38
SLCO2B1	solute carrier organic anion transporter family member 2B1	8	0.31	N/A	1	9	0.23
IL8	interleukin 8	9	0.31	624	1.43	90	0.44
ESM1	endothelial cell specific molecule 1	10	0.31	N/A	1	7	0.21
SERPINB2	serpin family B member 2	11	0.35	N/A	1	14	0.26
IL11	interleukin 11	12	0.36	N/A	1	24	0.33
CXCL5	C-X-C motif chemokine ligand 5	13	0.37	N/A	1	34	0.36
IL1RN	interleukin 1 receptor antagonist	14	0.37	N/A	1	26	0.33
CLDN14	claudin 14	15	0.38	N/A	1	72	0.42
BCL2A1	BCL2 related protein A1	16	0.38	N/A	1	51	0.40
PTGS2	prostaglandin-endoperoxide synthase 2	17	0.38	N/A	1	80	0.43
RPL24P2	ribosomal protein L24 pseudogene 2	18	0.39	N/A	1	365	0.60
IL1A	interleukin 1 alpha	19	0.39	599	1.39	81	0.43
CTD-2319I12.1	long non-coding RNA	20	0.40	N/A	1	77	0.42

Table S7: Top 20 up/down-regulated genes detected in IFN-β-pre-treated DAOY cells infected with TBEV compared to untreated mock cells. For completeness, other comparative data lists are also included. The rank (if possible, otherwise N/A – not available) represents the position of the gene in the list of differentially expressed genes of respective dataset; fold-change for each gene in all three datasets is shown.

up-regulated genes		mock vs TBEV+IFN-β		mock vs TBEV		mock vs IFN-β	
gene	Entrez name	rank	fold change	rank	fold change	rank	fold change
IFI27	interferon alpha inducible protein 27	1	44.67	75	2.48	1	32.53
IFI6	interferon alpha inducible protein 6	2	26.76	146	1.79	2	18.52
IFITM1	interferon induced transmembrane protein 1	3	12.76	N/A	1	6	9.21
BST2	bone marrow stromal cell antigen 2	4	5.09	174	1.68	8	3.8
RMRP	RNA component of mitochondrial RNA processing endoribonuclease	5	3.77	N/A	1	4	10.82
EPSTI1	epithelial stromal interaction 1 (breast)	6	3.52	38	3.56	9	2.72
KCNIP1	potassium voltage-gated channel interacting protein 1	7	3.51	N/A	1	N/A	1
HPGD	hydroxyprostaglandin dehydrogenase 15-(NAD)	8	3.27	N/A	1	N/A	1
CTD-2566J3.1	long non-coding RNA	9	3.17	N/A	1	N/A	1
GALNT15	polypeptide N-acetylgalactosaminyltransferase 15	10	3.08	N/A	1	N/A	1
RP5-1021I20.2	long non-coding RNA	11	3.00	N/A	1	10	2.66
RN7SK	RNA, 7SK small nuclear	12	2.89	N/A	1	5	9.63
IFITM3	interferon induced transmembrane protein 3	13	2.64	N/A	1	15	2.11
CRABP2	cellular retinoic acid binding protein 2	14	2.59	N/A	1	13	2.25
STMN2	stathmin 2	15	2.57	86	2.34	N/A	1
PCDP1	cilia and flagella associated protein 221	16	2.56	N/A	1	N/A	1
ISG15	ISG15 ubiquitin-like modifier	17	2.46	52	3.02	40	1.77
RGCC	regulator of cell cycle	18	2.46	N/A	1	17	2.07
TNFSF18	tumor necrosis factor superfamily member 18	19	2.38	91	2.30	34	1.79
RNA5-8SP6	RNA, 5.8S ribosomal pseudogene 6	20	2.36	836	0.51	3	12.58
**down-regulated genes**		**mock vs TBEV+IFN-β**		**mock vs TBEV**		**mock vs IFN-β**	
**gene**	**Entrez name**	**rank**	**fold change**	**rank**	**fold change**	**rank**	**fold change**
CTD-2328D6.1	long non-coding RNA	1	0.12	1	0.10	65	0.59
CCL3	C-C motif chemokine ligand 3	2	0.12	794	2.30	2	0.16
CSF3	colony stimulating factor 3	3	0.18	430	0.73	3	0.19
IL36RN	interleukin 36 receptor antagonist	4	0.19	N/A	1	1	0.12
RP3-483K16.4	long non-coding RNA	5	0.21	N/A	1	N/A	1
IGF2	insulin like growth factor 2	6	0.21	9	0.38	N/A	1
ESM1	endothelial cell specific molecule 1	7	0.21	N/A	1	10	0.31
AC069363.1	long non-coding RNA	8	0.22	814	2.55	N/A	1
SLCO2B1	solute carrier organic anion transporter family member 2B1	9	0.23	N/A	1	8	0.31
IL1B	interleukin 1 beta	10	0.23	N/A	1	4	0.22
MS4A7	membrane spanning 4-domains A7	11	0.24	N/A	1	N/A	1
RP11-907D1.2	long non-coding RNA	12	0.25	N/A	1	21	0.40
FAM71D	family with sequence similarity 71 member D	13	0.25	8	0.37	N/A	1
SERPINB2	serpin family B member 2	14	0.26	N/A	1	11	0.35
AC144831.1	long non-coding RNA	15	0.29	N/A	1	N/A	1
NOTCH3	NOTCH3	16	0.29	16	0.29	N/A	1
BCL9L	B cell CLL/lymphoma 9-like	17	0.30	5	0.36	N/A	1
ZNF469	zinc finger protein 469	18	0.30	64	0.52	96	0.68
COL1A1	collagen type I alpha 1	19	0.32	4	0.34	N/A	1
KDM6B	lysine demethylase 6B	20	0.32	N/A	1	N/A	1

Table S8: List of primers used for qRT-PCR analysis. Primer pairs marked (a) or (b) were obtained from either Primer bank [2] or RTprimerDB [3], respectively.

primer	sequence (5' - 3')	reference/primer ID
CCL3-F	CGGTGTCATCTTCCTAACCA	3599[b]
CCL3-R	GACATATTTCTGGACCCACTC	
CSF3-F	ATCTCCCCCGAGTTGGGTCC	
CSF3-R	TGGAAAGCAGAGGCGAAGGC	
CXCL10 F	GTGGCATTCAAGGAGTACCTC	
CXCL10 R	GGATTCAGACATCTCTTCTCACC	
CXCL11-F	GACGCTGTCTTTGCATAGGC	307611978c1[a]
CXCL11-R	GGATTTAGGCATCGTTGTCCTTT	
HPRT-F	TGACACTGGCAAAACAATGCA	[1]
HPRT-R	GGTCCTTTTCACCAGCAAGCT	
IFI27-F	GCTCTCACCTCATCAGCAGTGACC	
IFI27-R	CAAAACTACGGCAGAGCCAGAGG	
IFI6-F	GGTCTGCGATCCTGAATGGG	94538330c1[a]
IFI6-R	TCACTATCGAGATACTTGTGGGT	
IFIT2-F	GACACGGTTAAAGTGTGGAGG	
IFIT2-R	CAGACGGTAGCTTGCTATTGC	
IFNA-F	GTGAGGAAATACTTCCAAAGAATCAC	3541[b]
IFNA-R	TCTCATGATTTCTGCTCTGACAA	
IFNB1-F	CAGCAATTTTCAGTGTCAGAAGC	3542[b]
IFNB1-R	TCATCCTGTCCTTGAGGCAGT	
IFNG1-F	TCGGTAACTGACTTGAATGTCCA	56786137c1[a]
IFNG1-R	TCGCTTCCCTGTTTTAGCTGC	
IFNL1-F	GACCGTGGTGCTGGTGACTT	
IFNL1-R	AATGTGGCAGCCCTTCCCAG	
IFNAR1-F	CTCATTTACACCATTTCGCAAAGC	
IFNAR1-R	TCCAAAGCCCACATAACACTATC	
IFNAR2-F	ACCACTCCATTGTACCAACTCA	46488936c2[a]
IFNAR2-R	TGTGCTTCTCCACTCATCTGT	
IFNLR1-F	AGAATGTGACGCTGCTCTCC	
IFNLR1R	TTGGTTCCCGCACACTCTTC	
IL10RB-F	ACAACCCATGACGAAACGGT	
IL10RB-R	GGGGAGAAGGCGTACTTTGT	
OASL-F	CTGATGCAGGAACTGTATAGCAC	38016933c1[a]
OASL-R	CACAGCGTCTAGCACCTCTT	
RSAD2-F	CAAGACCGGGGAGAATACCTG	19923667a3[a]
RSAD2-R	GCGAGAATGTCCAAATACTCACC	

REFERENCES:

[1] **Vandesompele J, De Preter K, Pattyn F, et al.** Accurate normalization of real-time quantitative RT-PCR data by geometric averaging of multiple internal control genes. *Genome Biol* 2002; 3:RESEARCH0034.

[2] **Wang X, Seed B.** A PCR primer bank for quantitative gene expression analysis. *Nucleic Acids Res* 2003; 31:e154.

[3] **Pattyn F, Speleman F, De Paepe A, Vandesompele J.** RTPrimerDB: the real-time PCR primer and probe database. *Nucleic Acids Res* 2003; 31:122–3.

Fig. S1: DAOY and U373 cells express both neuronal and glial markers. DAOY and U373 cells were seeded on a chamber slide and incubated 24 hours. Cells were co-stained with antibodies for TUBB3 (green) and MOG (red). Representative pictures from two independent experiments performed in triplicates are shown. Scale bar represents 20 μm.

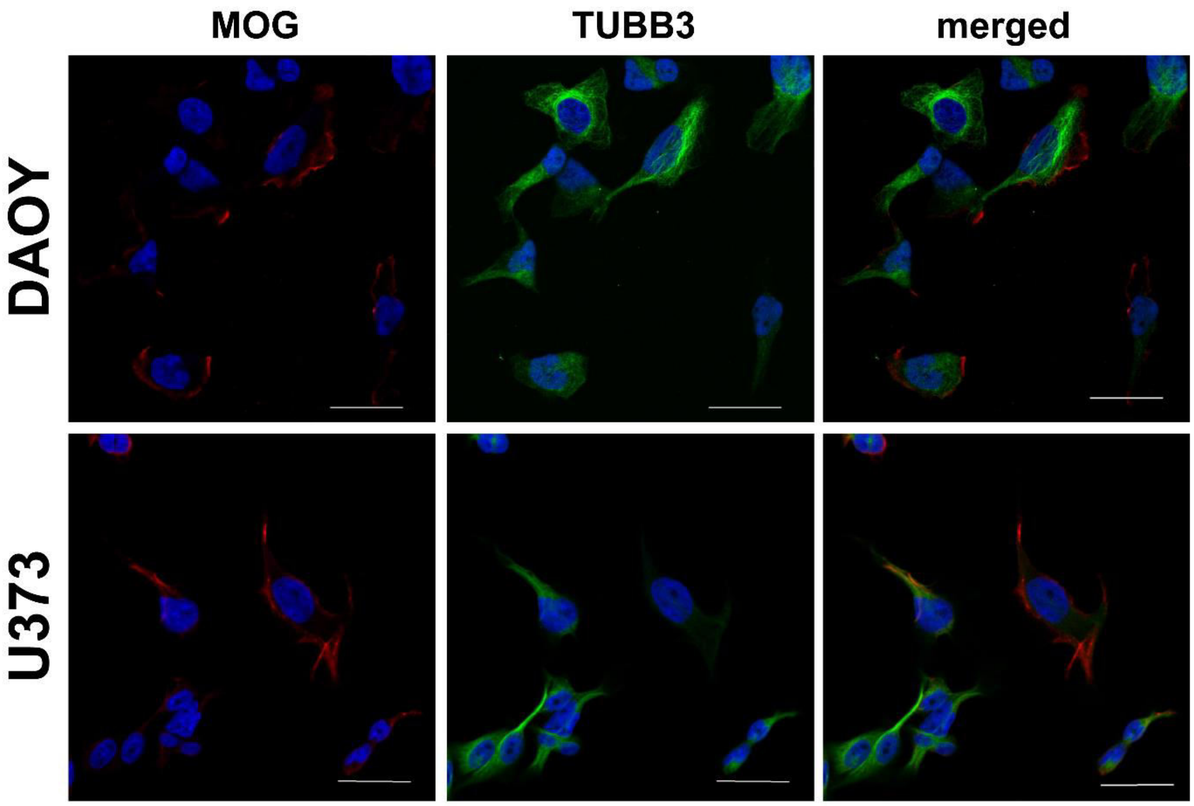

Fig. S2: Expression of neural markers is not affected by TBEV and does not define viral tropism. DAOY cells were seeded on a slide and infected at a MOI of 5 with TBEV Neudoerfl strain. Detection of viral NS3 protein and CNS markers by immunofluorescence was carried out at 24 hpi. Two independent experiments (performed with triplicates) were performed. **(a)** Percentage of cells expressing the respective marker in TBEV-infected or control cells is indicated. Average values and standard deviations are shown. Expression of TUBB3, MOG, and VIM was not significantly changed upon TBEV infection (Student‘s t-test; p=0.9679; p=0.9249; p=0.2244, respecitvely). **(b)** Percentage of TBEV-infected cells (NS3 positive) expressing the respective markers is presented. Average values and standard deviations are shown.

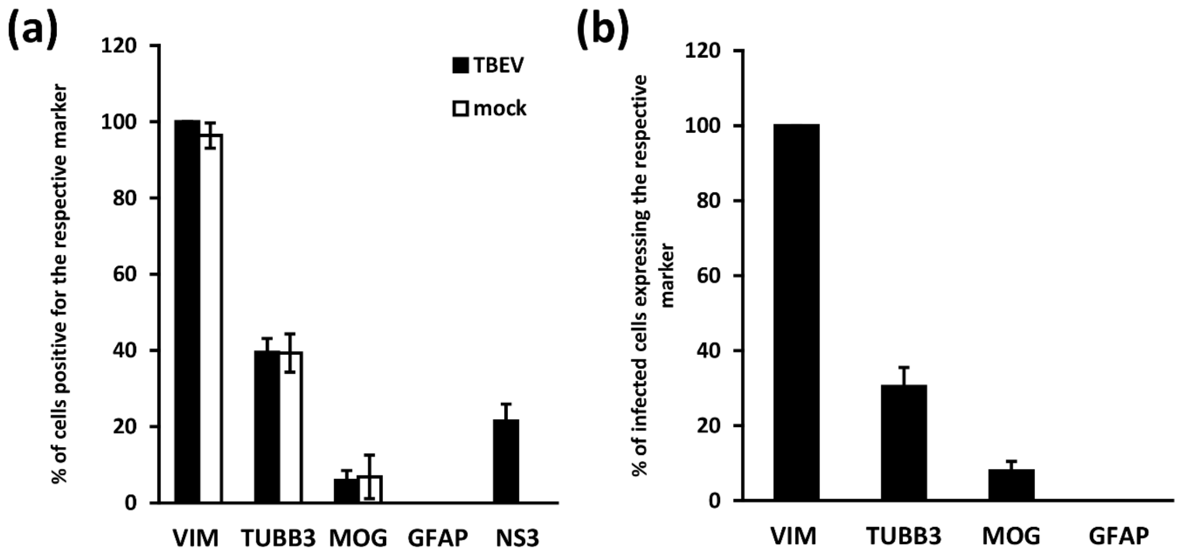

Supplement: Supplementary File 1 [file jgv-99-1147-s001.pdf]
